# Traits of litter‐dwelling forest arthropod predators and detritivores covary spatially with traits of their resources

**DOI:** 10.1002/ecy.2815

**Published:** 2019-08-14

**Authors:** Pierre‐Marc Brousseau, Dominique Gravel, I. Tanya Handa

**Affiliations:** ^1^ Département des Sciences Biologiques Université du Québec à Montréal Montréal Quebec H2X 1Y4 Canada; ^2^ Canada Research Chair in Integrative Ecology Département de Biologie Université de Sherbrooke Sherbrooke Quebec J1K 2X9 Canada

**Keywords:** co‐distribution, food web, functional traits, ground beetles, millipedes, Opiliones, spiders, trait matching, trophic interactions

## Abstract

The functional trait approach proposes that relating traits of organisms within a community to variation in abiotic and biotic characteristics of their environment will provide insight on the mechanisms of community assembly. As traits at a given trophic level might act as filters for the selection of traits at another trophic level, we hypothesized that traits of consumers and of their resources covary in space. We evaluated complementary predictions about top‐down (negative) and bottom‐up (positive) trait covariation in a detrital food web. Additionally, we tested whether positive trait covariation was better explained by the Resource Concentration Hypothesis (i.e., most commonly represented trait values attract abundant consumers) or the Resource Specialization Hypothesis (i.e., resource diversity increases niche availability for the consumers). Macroarthopods were collected with pitfall traps over two summers in three forested sites of southern Quebec in 110 plots that varied in tree species composition. Six feeding traits of consumers (detritivores and predators) and six palatability traits of their resources (leaf litter and prey) were matched to assess spatial covariation. Trait matches included consumer biting force/resource toughness, detritivore mandibular gape/leaf thickness, predator/prey body size ratio, etc. Our results demonstrate for the first time a covariation between feeding traits of detritivores and palatability traits of leaf litter (31–34%), and between feeding traits of litter‐dwelling predators and palatability traits of potential prey (38–44%). The observed positive covariation supports both the Resource Concentration Hypothesis and Resource Specialization Hypothesis. Spatial covariation of consumer and resource traits provides a new tool to partially predict the structure of the detrital food web. Nonetheless, top‐down regulation remains difficult to confirm. Further research on top‐down processes will be undoubtedly necessary to refine our capacity to interpret the effect of biotic interactions on co‐distribution.

## Introduction

Functional traits are morphological, physiological, phenological, or behavioral characteristics measurable at the individual level that can be related to the fitness of an organism (Violle et al. 2007, Pey et al. 2014). Abiotic and biotic environmental characteristics can act as filters selecting individuals based on these characteristics (Diamond 1975, Keddy 1992, Shipley 2010). Thus, relating functional trait composition of a community to environmental conditions could provide insight on the mechanisms of community assembly and allow for a more predictive ecology (McGill et al. 2006, Brousseau et al. 2018a). While considerable advances have been made in plant functional ecology (Violle et al. 2007, Reich 2014, Garnier et al. 2016), the use of a functional trait approach with animals has been slower to implement and much remains to be done (Pey et al. 2014, Moretti et al. 2017, Brousseau et al. 2018a). Considering animals in a multi‐trophic functional perspective complicates the identification of filters influencing the structure and function of communities, as traits at a given trophic level might act as filters for the selection of traits at another trophic level (Ibanez 2012, Lavorel et al. 2013, Le Provost et al. 2017). For instance, predator communities with particular feeding traits can select particular prey based on their palatability traits. Alternatively, palatability traits of prey can determine the available resources and drive which predators could be present in a community. As a consequence, when adopting a multi‐trophic community approach, one can hypothesize that traits should covary among consumers and their resources in both space and time, but that the direction may vary depending on whether top‐down or bottom‐up processes are more important within the ecosystem.

Food web structure has been shown to be associated with different aspects of ecosystem functioning (Bartomeus et al. 2016, Gravel et al. 2016), but resolving complex food webs is time consuming and often beyond reach. Tools that allow us to extrapolate the effects of food web structure on ecosystem function are needed. One way to move forward without resolving entire food webs involves matching the consumer feeding traits and resource palatability traits (i.e., traits determining the degree to which a resource is edible) that determine trophic interactions. Trait matches that predict interactions can be validated through lab arena experiments (e.g., Brousseau et al. 2018b) or through models analyzing well‐resolved food webs (e.g., Laigle et al. 2018). Examples of trait matching include the length of pollinator tongue and corolla depth of flowers (Ibanez 2012), biting force of ground beetles and cuticular toughness of prey (Brousseau et al. 2018b) and lipid content of predatory marine mammals and caloric content of prey (Spitz et al. 2014). A match of traits between the consumer and the resource is the first condition for an interaction to occur (Bartomeus et al. 2016, Schmitz 2017). A second condition is that species must encounter each other in space and time, i.e., they must share similar traits to pass through the same spatiotemporal and abiotic filters (Gravel et al. 2016). However, given the potential for trait convergence (e.g., traits related to resistance to desiccation) in situations where abiotic filtering is much stronger than biotic filtering, mismatches can also result between consumer feeding traits and the palatability traits of their resources (Le Provost et al. 2017).

Complementary hypotheses can be postulated for how traits will covary over space and time and whether assembly follows a bottom‐up or a top‐down sequence when biotic filters are at play. (1) Following the Resource Concentration Hypothesis (Root 1973), it can be hypothesized that the most commonly represented resource trait values (expressed as the community weighted mean, CWM) will attract abundant consumers with matching traits (Le Provost et al. 2017). Thus, a forest dominated by trees that produce tougher leaf litter should favor detritivores with a stronger biting force. (2) The Resource Specialization Hypothesis (Southwood et al. 1979) proposes that diversity of resource palatability traits (expressed as functional dispersion, FDis) can increase niche availability for consumers, which will be reflected in an increase of the FDis of their matching feeding traits (Gravel et al. 2016). Thus, if leaves found in the litter community vary greatly in toughness, the leaf litter should sustain a detritivore community with a high variability in their biting force. For both these bottom‐up hypotheses, we predicted a positive covariation of trait matches as trait values of resources will favor consumers harboring matching trait values.

Predictions associated with top‐down control are less clear‐cut, since increasing consumer functional diversity might enhance resource consumption but increasing resource functional diversity might counteract this response (Gravel et al. 2016). For example, a detritivore community composed mainly of species with weak mandibles could rapidly consume the least tough leaves from the previous autumn, leaving a litter composed mainly of tough leaves. Here, we predicted that (3) top‐down pressure will be higher on resources whose traits match the feeding traits of consumers resulting in a negative covariation between traits of consumers and resources. These bottom‐up and top‐down hypotheses have never been tested with explicit trait matches of consumers and resources in soil ecosystems, but Milcu et al. (2013) found that the relation between the functional diversity of plants and detritivores was better explained by the Resource Specialization Hypothesis in a soil grassland ecosystem.

Our study aimed to test the general hypothesis that resource palatability traits will covary in space with associated consumer feeding traits using empirical data from three distinct forest communities of litter‐dwelling macroarthropod detritivores and predators and their respective leaf litter or prey resources. We expected that covariation between consumer and resource traits will follow a bottom‐up sequence and will be stronger when considering FDis (which better reflects niche diversity) than CWM. Macroarthropods were sampled over two summers in three different forest sites that varied substantially in overstory tree composition and thus leaf litter. We analyzed the relationship between feeding traits and palatability traits of detritivores and leaf litter, and of invertebrate predators and prey based on three trait matches (Table 1) to determine if they covaried in space.

**Table 1:** List of hypothetical trait matches between feeding traits of consumers (detritivores or predators) and palatability traits of their resources (leaf litter or prey)

Trait matches	Hypotheses
Detritivores ↔ leaf litter	
Biting force ↔ toughness	Strength is related to the toughness of interacting litter (Ibanez et al. 2013).
Mandibular gape ↔ thickness	Leaf thickness limits manipulation ability based on mandibular gape (Bernays 1998).
Strength of mandibular molar plate ↔ dry matter content	The molar plate is mainly used to crush food particles and extract water (Köhler and Alberti 1990); stronger pressing could be required to extract water from leaves with high dry matter content.
Predators ↔ prey	
Body volume ↔ body volume	Body size ratio is a determinant of predator–prey interactions (Gravel et al. 2013).
Biting force ↔ cuticular toughness	Strength is related to the toughness of interacting prey (Brousseau et al. 2018b).
Mechanical advantage ↔ body width	Mechanical advantage of predator mouthparts (length : width ratio) reflecting the handling ability of food having different shapes (Evans and Forsythe 1985) can be related to prey body width.

## Methods

### Study sites

Sampling was carried out in three protected temperate deciduous forests of southern Quebec, Canada (Parc national du Mont St. Bruno, Gault Nature Reserve of Mont St. Hilaire, and Mont Écho in the Montagnes Vertes ecological reserve). Sites at Mont St. Bruno and Mont St. Hilaire are separated by ~12.5 km while Mont Écho is ~75 km south of them. Mont St. Bruno (45°33′09″ N, 73°19′18″ W) and Mont St. Hilaire (45°32′59″ N 73°09′39″ W) are situated in the St. Lawrence Lowland and are dominated by deciduous forests surrounded by agricultural and suburban developments. The forest is mainly composed of *Acer saccharum*,* Quercus rubra*, and *Fagus grandifolia*. Other common trees in these sites include *Acer pensylvanicum*,* Fraxinus americana*, and *Ostrya virginiana*. Mont Écho (45°06′06″ N, 72°30′37″ W) is part of the Appalachian Mountains and is dominated by mature forest of *A. saccharum* and *F. grandifolia* at low elevation, and *Abies balsamea* and *Betula* spp. at higher elevation. Some sections of the study site are also covered by high densities of the shrub *Viburnum lantanoides* and ferns (mainly *Dennstaedtia punctilobula*). Average annual temperature from 2000 to 2012 was 7°C and 6°C, respectively, at Mont St. Bruno/Mont St. Hilaire and Mont Écho with average annual precipitation of 1,071 and 1,371 mm (Environment Canada 2016). Total precipitation during the 2011 and 2012 sampling period was respectively 331 and 211 mm at Mont St. Bruno/Mont St. Hilaire, and 496 and 303 mm at Mont Écho (Environment Canada 2016).

### Sampling plots

Ten sampling plots were installed at both Mont St. Hilaire and Mont St. Bruno, in three distinct stands dominated by *A. saccharum*,* Q. rubra*, or *F. grandifolia* for a total of 30 plots (1 m radius, centered on a pitfall trap) per site. Plots were at least 40 m apart. A Hobo data logger (Onset Computer Corporation, Bourne, MA, USA) was installed at 1 m height in each plot to record temperature hourly between 23 June and 30 August 2012. Soil humidity was measured at the center of the plot at each sampling periods in 2012 with a Field Scout TDR 300 (Spectrum Technologies, Inc., Aurora, IL, USA) with 4 cm long rods. Altitudinal range at these sites was 203–272 m and 105–183 m, respectively.

Fifty plots were installed at Mont Écho along two parallel transects (25 plots/transect) covering an elevation gradient of 200 m (altitudinal range was 572–769 m) across the gradient between the *A. saccharum* and the *A. balsamea* stands. Plots were separated along transects by 40 m. Temperature and humidity were recorded as at Mont St. Bruno/Mont St. Hilaire.

### Arthropod sampling

One Multi‐Pher pitfall trap (Jobin and Coulombe 1988) was installed at each of the 110 plots. Traps were continuously active from mid June to late August 2011, and samples were collected every 2–3 weeks. In the following year, traps were active only during two consecutive weeks in June, July, and August. Pitfall traps were 12 cm in diameter and 16 cm depth. Ethyl alcohol (40%) with 5% white vinegar was used for preservation.

### Vegetation characterization and functional traits

Litter samples were collected adjacent to each pitfall trap (<1 m away) once in August 2011, and once during each trapping period in 2012. These samples consisted primarily of partially decomposed deciduous leaves and senesced evergreen needles. Sample volume was standardized as the volume that filled a 20.4 × 15.4 cm plastic bag without compaction. All leaves were visually identified to species when possible and individually weighed to the nearest 0.01 g.

Three functional traits, related to leaf litter decomposability and palatability for detritivore arthropods (see justification in *Arthropod identification and functional traits*), were measured: leaf toughness, leaf thickness, and leaf dry matter content (Bernays 1998, Makkonen et al. 2012, David 2014). All traits were measured on 10 leaves selected randomly, avoiding recently fallen (partly green leaves), across samples (or the maximum number available) per species per stand at Mont St Bruno/Mont St. Hilaire, and from five evenly distributed distance intervals (0–180, 200–380, 400–580, 600–780, 800–980 m) at Mont Écho. Litter was rehydrated before measuring traits by gradually spraying water on leaves in a closed plastic box until saturation was reached, i.e., when water droplets accumulated at the leaf surface and in the tray. Leaf toughness (g/mm^2^), defined as the pressure required to perforate the leaf, was measured with a Pesola Medio‐Line pressure set (Pesola Präzisionswaagen AG, Schindellegi, Switzerland). Up to five toughness measurements were taken on each of 10 leaves, and the average value per leaf was used in the analysis. Leaf thickness was measured with a microcaliper to the nearest μm. Leaf dry matter content was measured as the mass of the dry leaf (at 65°C) divided by the mass of the hydrated leaf (measured after the rehydration process).

### Arthropod identification and functional traits

All Diplopoda, Isopoda, Carabidae (adults and larvae), Araneae, and Opiliones caught in pitfall traps were identified to the species level. Larvae of Diptera, Coleoptera, Mecoptera, and Lepidoptera were identified as morphospecies.

The traits of consumers and resources were selected to represent three potential feeding trait matches for each trophic interaction (detritivore/litter and predator/prey; Table 1). For detritivore/litter interactions, we also included detritivore body volume as an unmatched trait to represent different aspects of feeding (Brown et al. 2004). Body volume (mm^3^) was evaluated based on shape, length, width, and height of each species and used as a measure of body size. Cuticular toughness was measured using a Pesola Medio‐Line pressure set to which we added an entomological pin of size 2 (diameter = 0.45 mm). Toughness, in g/mm^2^, was defined as the pressure required to break through the integument with the pin. A value of zero was given for very small and soft species while a value of 1 was given to small, but sclerotized, species. A biting force index at mouthpart tip was measured based on the formula *h* × *b*/*c*, where for mandibulate arthropods, *h* is the width of the head behind the eyes (i.e., the attachment point of the adductor muscle of the mandibles), *b* is the basal width of the mandible between the upper condyle and the insertion point of the adductor muscle, and *c* is the length from the upper condyle to tip (Wheater and Evans 1989). For arachnids, *h* is the size (length × width) of left chelicera, *b* is the basal width of the movable digit, and *c* is its length (van der Meijden et al. 2010). The biting force index of the molar plate was evaluated by measuring *h* as the length of the stipe, *b* between the condyle and the insertion point of the abductor muscle, and *c* between the condyle and the midpoint of the molar plate. The traits were measured on at least six specimens per species (or the number of available specimens). Complete protocols for the measurement of arthropod traits are available in Appendix S1.

### Statistical analysis

Several pitfall trap samples were lost due to mammal disturbance or flooding, resulting in an unequal number of days per trap between plots. We did not analyze data from six plots that were active for less than two‐thirds of the trapping periods (Table 2). The abundance of each species was summed in each plot and corrected to reflect a total of 85 d of trapping by applying the formula 85 × abundance/trapping days, rounded to the upper whole number. The detritivore guild included Diplopoda and Isopoda, while the predator guild included Araneae, Opiliones, and Carabidae (adults and larvae). Prey included detritivores and larvae (excluding Carabidae). The very large millipede species *Narceus americanus* was not considered as a prey as it is unlikely to be predated by any of observed predators and would have artificially inflated the CWM and FDis of prey where the species is present.

**Table 2:** Summary description of the communities at each sampling site for each trophic level

Component	Mont St. Bruno	Mont St. Hilaire	Mont Écho	All
Plot (no.)	27	29	48	104
Humidity min–max (%)^a^	3–14	4–17.2	4.5–37.7	
Temperature min–max (°C)^a^	20.7–21.4	19.9–20.8	16.4–19.1	
Plant detritus				
Leaf litter species richness	8	6	9	13
Prey				
Detritivores Diplopoda				
Abundance	112 ± 15	118 ± 9	17 ± 1	7,270
Richness	15	9	6	16
Isopoda				
Abundance	71 ± 9	7 ± 1	0	2,121
Richness	4	3	0	4
Larvae				
Abundance	22 ± 3	19 ± 1	16 ± 1	1,887
Richness	57	67	84	115
Predators				
Carabidae				
Abundance	190 ± 19	146 ± 11	109 ± 8	14,635
Richness	22	26	32	46
Araneae				
Abundance	46 ± 5	82 ± 5	71 ± 4	6,963
Richness	58	56	71	115
Opiliones				
Abundance	25 ± 5	11 ± 1	7 ± 1	1,328
Richness	10	11	9	16

Notes

Diplopoda and Isopoda were considered both detritivores (consuming leaf litter) and prey (to predators). Abundance is presented as the mean (± SE) per plot and richness as the total number of species per site. Min, minimum; max, maximum.

a Mean per plot.

The first objective in the analyses was to determine if (and which) environmental factors influenced the structure of detritivore and predator communities and the distribution of their feeding traits. To answer this question, we first described resource community structure with a principal component analysis (PCA). A PCA was performed for each resource (leaf litter and prey) on the abundance of the species in Hellinger distance (Legendre and Gallagher 2001). The PCA scores were then used as explanatory variables for a redundancy analysis (RDA) of detritivore and predator communities. The rotation of leaf litter and prey species is presented in Appendix S2: Figs. S1–S3.

For each consumer level (detritivores and predators), we performed three RDAs on the species abundance in Hellinger distance, and on standardized CWM and FDis of their feeding traits per plots. The environmental matrix included temperature, humidity, and the species structure (based on the PCA scores) and functional structure of the lower trophic level. For detritivores, the scores of axes explaining more than 10% of the observed variation of leaf litter (i.e., the first three axes) were included. Functional structure was represented as the CWM of all three leaf palatability traits. The same logic was used for predator communities, but the PCA scores on prey species were added, as well as the PCA scores on leaf litter species, which can influence shelter and hunting field of predators (Vehviläinen et al. 2008). Three prey axes were used to be symmetric with leaf litter despite the third PCA axis explaining < 10% of the variation. Also, the CWM of the palatability traits of leaf litter was replaced by those of the prey. All explanatory variables were standardized. The significant contribution of explanatory variables was assessed with permutation tests with 9,999 iterations using the function anova in R (R Core Team 2017). Using the CWM, the FDis or both metrics to represent the functional structure of the resource in the RDA analysis yielded very similar results. For this reason, we only present results based on the CWM of resource traits.

The second objective of our analyses was to determine if consumer feeding traits and resource palatability traits covaried in space. In the case of positive covariation, we sought to evaluate if the covariation corresponded to the Resource Concentration Hypothesis or the Resource Specialization Hypothesis; i.e., determining if the covariation is better observed with the CWM or the FDis of traits. Finally, we evaluated how well trait covariation described consumer/resource distribution as compared to species composition. The covariation of consumer and resource traits was analyzed in a multivariate space with the Procrustes co‐inertia method (Legendre and Legendre 2012). Procrustes analyzes were also used to compare the species composition of each trophic level. We first performed PCA on each standardized functional component (CWM and FDis) and species abundance (in Hellinger distance) of the leaf litter, detritivores, prey, and predators independently. The PCAs were then compared (leaf litter vs. detritivores, and prey vs. predators) with a Procrustes analysis with the function protest of the vegan library in R with 9,999 iterations (Oksanen et al. 2013).

Procrustes analysis determines the degree of association between two matrices by scaling and rotating the data to find the maximum fit; the less that data are scaled and rotated, the greater the association between the matrices. Procrustes analyzes also allow to represent visually the concordance between the matrices (Peres‐Neto and Jackson 2001) and thus allows to interpret the degree and the direction of the covariation between traits of the compared trophic levels. If both traits of a match (e.g., detritivore mandibular strength and litter toughness) covary positively (e.g., the angle between the traits is small), it means that detritivores with stronger mandibles are found in sites characterized by tough litter. In light of the hypotheses presented here, this scenario would be better explained by a bottom‐up control since top‐down regulation would decrease resource availability. Thus, top‐down control is expected to yield a negative covariation of these traits: i.e., the detritivores with weaker mandibles are characteristic of sites characterized by tough litter as the relative abundance of weak litter will decrease following its consumption.

## Results

### Species diversity

Overall, we identified 13 plant, 20 detritivore, 177 predator, and 115 prey larva (from which 62 of 115 were caught fewer than five times) species (Table 2 and Appendix S3). Species richness of detritivores was higher at Mont St. Bruno (19 species) than at Mont St. Hilaire (12 species) and Mont Écho (six species). Carabidae were the most abundant predator taxa with 14,635 specimens caught, but Araneae had the highest species richness with 115 species (Table 2). Species richness of Araneae was higher at Mont Écho than in other sites and their abundance was particularly low in Mont St. Bruno. Inversely, Carabidae and Opiliones were more abundant at Mont St. Bruno than in other sites, but the richness of Carabidae was higher at Mont Écho where 32 species were observed.

Species composition of leaf litter and prey species varied across sites, particularly at Mont Écho, as reflected in the PCA analysis (Appendix S2: Fig S4). Leaf litter was representative of dominant tree species with only *A. saccharum* and *F. grandifolia* common to all three sites (Appendix S2: Fig. S4A and Appendix S3: Tables S1). The presence of *A. balsamea* and *Betula* spp. separated Mont Écho from the other sites (Appendix S2: Fig. S4A). Litter composition was more similar across sites on axes 2 and 3 (Appendix S2: Fig. S4B). The first three axes of the leaf litter PCA explained respectively 56%, 16%, and 14% of the total variation. The PCA of prey species distribution revealed a separation of Mont Écho from other sites on the first axis, and a separation of Mont St. Bruno and Mont St. Hilaire on the second axis (Appendix S2: Fig. S4C). All sites were similar on the third axis (Appendix S2: Fig. S4D). The first three axes of the PCA explained respectively 28%, 20%, and 6% of the overall variation in prey community composition.

### Detritivores: environmental factors and feeding traits

**Figure 1:**
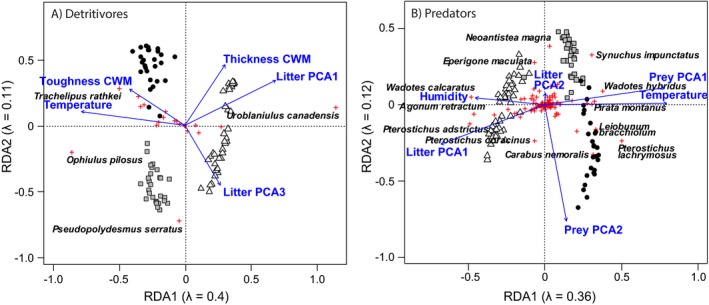
Redundancy analysis on the distribution of (A) detritivorous and (B) predatory arthropod species of forest litter sampled in three sites in southern Quebec: circle, Mont St. Bruno; square, Mont St. Hilaire; triangle, Mont Écho. Blue arrows indicate significant variables based on PERMANOVA.

**Figure 2:**
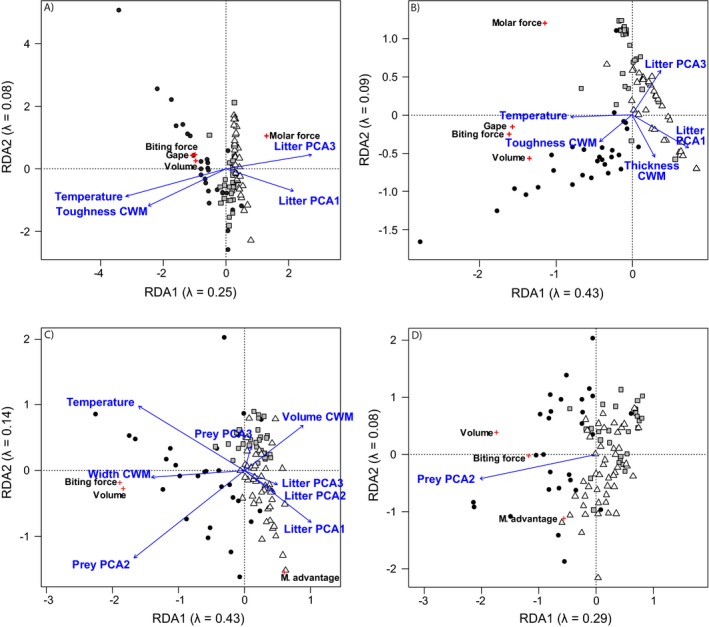
Representation of RDA on the distribution of feeding traits of (A, B) detritivorous and (C, D) predatory arthropods of forest leaf litter sampled in three sites in southern Quebec based on their (A–C) community weight mean (CWM) and (B–D) functional dispersion (FDis): circle, Mont St. Bruno; square, Mont St. Hilaire; triangle, Mont Écho. Blue arrows indicate significant variables based on PERMANOVA. M. advantage, mechanical advantage.

Communities of arthropod detritivores were well separated on the first two axes of the RDA showing distinct communities at the three sites (Fig. 1A). The first two axes explained respectively 42% and 12% of the observed variation in species composition. The variation explained by the first two axes was similar when analyzing the FDis (43% and 9%) but was lower when analyzing the CWM (25% and 8%). The separation of the sites was less clear for CWM and the FDis, but plots at Mont St. Bruno still tended to be distinct from the other two sites (Figs. 2A, B). Leaf litter community structure explained the most variation in the distribution of detritivore feeding traits (expressed as CWM or FDis; Table 3). Temperature, leaf litter toughness, and leaf litter thickness (only for FDis) also significantly explained feeding trait distribution of detritivores. All explanatory variables except humidity had a significant influence on detritivore distribution, but temperature contributed the most (Table 3).

**Table 3:** Summary statistics (*F* values and statistical significance, degrees of freedom: 1, 95) of redundancy analysis (RDA) performed on detritivore communities

Explanatory variables	Species	CWM	FDis
Humidity	2.3	0.5	0.4
Temperature	18.3***	6.2**	6.4**
Leaf litter community (PCA1)	6.9***	7.1**	11***
Leaf litter community (PCA2)	2.6*	0.2	0.2
Leaf litter community (PCA3)	3.8*	12.4***	12.1***
Leaf litter thickness CWM	2.8*	1.2	3.8*
Leaf litter toughness CWM	2.6*	4.4*	6.6**
Leaf dry matter content CWM	2.6*	0.2	1.8
Total variation explained	0.57	0.33	0.52

Species composition, community weighted mean (CWM) and functional dispersion (FDis) of four feeding traits (body volume, mandibular gape, biting force at the tip of the mandibles, and biting force of the molar plate of the mandibles) were compared. A significant value indicates that the variables significantly explain a part of the variation observed in the species or functional structure of the community.

*P ≤ 0.05; **P ≤ 0.01; ***P ≤ 0.001.

### Detritivore–litter: covariation in multivariate space

**Figure 3:**
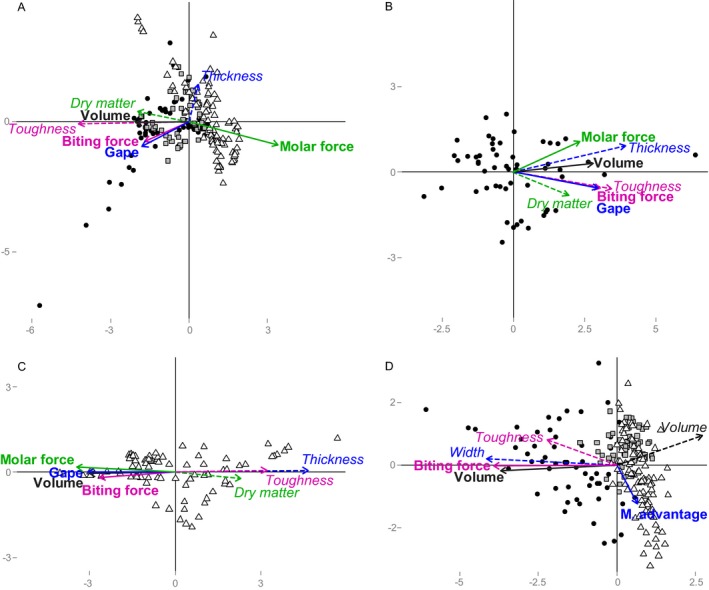
Procrustes analyses on the community weighted mean (CWM) of palatability (in italic type with dashed arrow) and feeding traits (in boldface type with solid arrows) of (A–C) leaf litter and arthropod detritivores, or (D) potential prey and predators. Different colors indicate hypothesized trait matches. Circle, Mont St. Bruno; square, Mont St. Hilaire; triangle, Mont Écho. M. advantage, mechanical advantage

**Figure 4:**
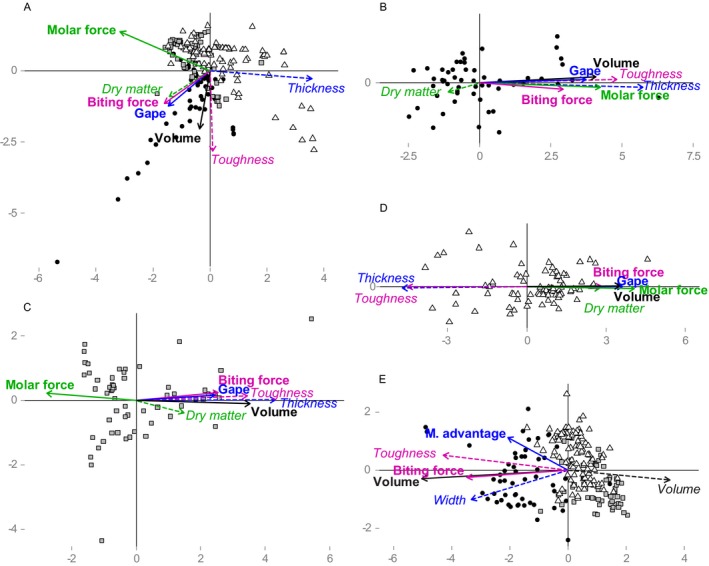
Procrustes analyses on the functional dispersion (FDis) of palatability traits of leaf litter (in italic type with dashed arrow) and feeding traits (in boldface type with solid arrows) of (A–D) leaf litter and arthropod detritivores, or (E) potential prey and predators. Different colors indicate hypothesized trait matches. Circle, Mont St. Bruno; square, Mont St. Hilaire; triangle, Mont Écho. M. advatange, mechanical advantage.

All Procrustes analyses between arthropod detritivore communities and leaf litter communities revealed significant correlations except for the CWM at Mont St. Hilaire (Table 4). At Mont St. Hilaire and Mont St. Bruno, palatability and feeding traits positively covaried following our trait match hypotheses (Table 1), i.e., detritivore biting force with leaf litter toughness, and detritivore mandibular gape with leaf litter thickness (Figs. 3B and 4B, C). At Mont Écho, however, all detritivore traits covaried negatively with all leaf litter traits, except dry matter content in FDis (Figs. 3C and 4D). Detritivore body volume positively covaried consistently with leaf litter toughness for both CWM and FDis, except at Mont Écho (Figs. 3 and 4). Procrustes analyzes consistently showed a higher correlation between FDis of palatability traits of leaf litter communities and feeding traits of arthropod detritivore communities than between CWM and these variables, but differences were not always clear between indices (Table 4). Correlations were generally highest when species composition was analyzed except at Mont St. Bruno. Results were similar when correlations were tested for each year separately, although the correlations tended to be lower in intrasite comparisons in 2012 (data not shown).

**Table 4:** Correlations based on Procrustes analyses of arthropod detritivore and leaf litter communities and arthropod predator and prey communities

Site	Species	CWM	FDis
Detritivores–litter			
All sites	0.6***	0.31***	0.34***
Mont St. Bruno	0.45*	0.41*	0.47**
Mont St. Hilaire	0.54**	0.26	0.35*
Mont Écho	0.61***	0.36**	0.4**
Predators–prey			
All sites	0.81***	0.38***	0.44***
Mont St. Bruno	0.77***	0.37	0.3
Mont St. Hilaire	0.83***	0.25	0.3
Mont Écho	0.8***	0.1	0.27

Note

Correlations were calculated for PCA of species structure or for functional structure indices (community weighted mean and functional dispersion) of contrasted trophic levels.

*P ≤ 0.05; **P ≤ 0.01; ***P ≤ 0.001.

### Predators: environmental factors and feeding traits

As observed for detritivores, predator species composition was distinct among the three sites (Fig. 1B), but sites were more similar in functional composition (Figs. 2C, D). The first two axes of the RDA on predator species composition explained 35% and 11%, respectively, of the observed variation. The variation explained by the first two axes was higher when analyzing the CWM (42% and 10%) but lower when analyzing the FDis (26% and 10%). Prey community structure had the highest influence on the variation in the distribution of the predator feeding traits expressed as CWM (Table 5). Temperature, litter community structure and the CWM of prey body volume and width also had a significant influence. In contrast, FDis of predator feeding traits was significantly explained only by the second PCA axis of prey community structure (Table 5). Prey community structure had the highest influence on the variation in the distribution of the predator species. Soil humidity and temperature, and the litter community structure also had a significant influence (Table 5).

**Table 5:** Summary statistics (*F* value and statistical significance, degrees of freedom: 1, 92) of redundancy analysis (RDA) performed on predator communities

Explanatory variables	Species	CWM	FDis
Humidity	2.3*	1.1	1.2
Temperature	2.4*	7**	1
Leaf litter community (PCA1)	2.9**	5.7**	1.5
Leaf litter community (PCA2)	2.4*	6.1**	2.3
Leaf litter community (PCA3)	1.8	3.9*	1.1
Prey community (PCA1)	2.4*	2	1.8
Prey community (PCA2)	6.4***	13.9***	7.8***
Prey community (PCA3)	1.3	4*	0.4
Prey volume (CWM)	1.7	4.1*	0.6
Prey width (CWM)	1.5	9***	2.6
Prey toughness (CWM)	0.8	1.1	0.8
Total variation explained	0.56	0.57	0.41

Species composition, community weighted mean (CWM), and functional dispersion (FDis) of three feeding traits (body volume, biting force, and the mechanical advantage (length : width ratio) of the mandibles) are compared. A significant value indicates that the variables significantly explain a part of the variation observed in the species or functional structure of the community.

*P ≤ 0.05; **P ≤ 0.01; ***P ≤ 0.001.

### Predators–prey: covariation in multivariate space

The Procrustes analysis on the CWM and FDis of prey traits and predator traits was significant only when all sites were analyzed together (Table 4). Predator biting force consistently covaried positively with prey cuticular toughness, and predator body volume consistently covaried with prey body width (Figs. 3D and 4E). In contrast, predator and prey body volume consistently covaried negatively. The correlation between species composition of arthropod predator and prey communities was always higher (Procrustes *R* ^2^ > 0.7) than for functional composition. The correlation was generally slightly higher for FDis than CWM except at Mont St. Bruno, where it was slightly lower. These results were also maintained when 74 additional prey taxa (sampled through Tullgren extractions and consisting of smaller body‐sized individuals) were included in the overall analyses (data not shown). The results also remained similar when correlations were tested for each year separately (data not shown).

## Discussion

Our results show a covariation between consumer (detritivores and predators) feeding traits and resource (leaf litter and prey) palatability traits in the leaf litter layer of deciduous temperate forests in southern Quebec. Most hypothesized trait matches covaried positively, indicating a bottom‐up sequence in most of the studied communities. These results help to better understand the role of traits in the structure of complex food webs and particularly inform us on connectance (i.e., the percentage of potential links realized) within the studied arthropod community. In a community with high trait covariation between consumer feeding and resource palatability, the probability that two encountering individuals harbor matching traits increases and results in greater connectance (see mathematical demonstration in Gravel et al. 2016). In detritivore/leaf litter interactions, such connectance could result in higher decomposition rates, while for predator–prey interactions, it could favor trophic regulation. Empirical data linking network connectance to ecosystem functions are still lacking, but our results suggest that soil food webs present an interesting model to test this link.

Our results also show that while the covariation between the feeding and palatability traits of consumers and their resources was generally better explained by the Resource Specialization Hypothesis (Table 4), the minimal differences in explanatory power make it impossible to accept this hypothesis over the Resource Concentration Hypothesis. Finally, the variation in species composition, functional composition (expressed as the CWM) and functional variance (expressed as the FDis) of arthropod detritivore communities is best explained by the same factors, but in different proportions (Table 3). Thus, microsite variation in temperature best described species composition, but the functional characteristics of arthropod detritivore communities were better explained by leaf litter species and functional structure. The results were not as clear for arthropod predator species, but prey species composition and functional composition explained a higher proportion of the functional structure of predators than abiotic condition and leaf litter community (Table 5).

### Trait matching

Simple feeding and palatability traits explained 31–44% of community structure of detritivorous and predatory macroarthropods dwelling in the forest leaf litter (Table 4). Our study included a high species richness of arthropods (312 species) and three sites presenting different species composition. In this context, our results suggest that as few as three functional trait matches per trophic level may be useful to consider biotic interactions at play in community assembly. Nonetheless, we recognize that our study did not consider other sources of variation that most likely contribute to explaining spatial covariation. For example, we only considered feeding interactions, while abiotic filters also play an important role in structuring forest litter‐dwelling arthropod communities (Frouz et al. 2004). While including traits related to abiotic factors may have increased our predictive ability, for a large diversity of arthropods, such traits that are related empirically to abiotic gradients are lacking (Moretti et al. 2017). We also did not include potential variation in resources present in each site such as intraspecific variation related to different decomposition stages or parts of plants, or potential prey excluded from the study, but present in the study system, such as earthworms, springtails, mites, nematodes, protists, and microbes. As the amplitude of seasonal functional variation was never studied for feeding and palatability traits of litter‐dwelling arthropods, it is unclear how seasonality, including phenology and developmental stages of arthropods, could influence our results. Finally, traits related to stoichiometry were shown to be useful to describe herbivore/ plant interactions (Deraison et al. 2015) and to be good predictors of detritivore–litter interactions in aquatic systems (Ohta et al. 2016). Including stoichiometry in future work will undoubtedly be interesting to explore, in addition to the physical aspects of feeding interactions studied here.

Our results show that the hypothesized trait matches between consumers and resources (Table 1) tend to covary in space for detritivore–litter and predator–prey interactions. We observed a consistent positive covariation between leaf litter toughness and detritivore biting force, except at Mont Écho (Figs. 3 and 4). Leaf litter thickness and detritivore mandibular gape also covaried positively at Mont St. Hilaire and Mont St. Bruno. Similar trait matching was observed for predators and prey (Figs. 3 and 4), although absence of significant intrasite collinearity did not allow for analysis of intrasite variation. Predator biting force covaried positively with prey cuticular toughness, and predator body volume covaried positively with prey body width. We also observed a negative covariation between the body volume of predators and prey. Based on our initial prediction, negative covariation was expected in the presence of top‐down control because of stronger pressure on prey with traits matching the feeding traits of predators. Our results could be interpreted as bottom‐up and top‐down processes acting simultaneously on different traits; i.e., predator body volume could have a top‐down effect on prey body volume while prey cuticular toughness has a bottom‐up effect on biting force of the predators. Alternatively, this may also result from body size being related to a high number of functions (Peters 1986), and thus being more sensitive to abiotic conditions, contrary to traits that are directly related to feeding such as biting force.

The negative covariation of body volumes vs. the positive covariation of other predator/ prey trait matches has another alternative explanation. The coevolutionary alternation hypothesis (Thompson 1999) predicts that predation pressure will trigger a higher display of defense in more commonly attacked prey, prompting the predator to include new prey that are less defended in its diet. The predation release will then induce a reduction of defense due to metabolic costs creating an interaction loop (Nuismer and Thompson 2006). If this hypothesis is right, we should expect an alternation in the direction of the covariation of some matching traits through time. In our case, we could expect that body volume of predators and prey would covary positively and the strength of predators and toughness of the prey would covary negatively. It is unclear on what timescale such micro‐coevolution can be observed, but it could require several decades (Thompson 1999). Such variation should be easier to observe by monitoring defensive traits of the prey such as chemical and physical defense.

The importance of trait matching to explain food web interactions has been demonstrated previously for arthropods (Ibanez 2012, Ibanez et al. 2013, Deraison et al. 2015, Brousseau et al. 2018b), but has been rarely used to explain community structure. Nonetheless, spatial covariation was previously observed between grasshopper community biting force and plant community leaf toughness (Le Provost et al. 2017), pollinator tongue length and flower corolla depth (Garibaldi et al. 2015) and bird beak size and fruit size (Dehling et al. 2014). Similar covariation was observed between feeding and palatability traits of zoo‐ and phytoplankton in boreal lakes, although this covariation was no longer significant when abiotic and landscape filtering were considered (St‐Gelais et al. 2018). Our study is the first to use trait matching to investigate community structure of litter‐dwelling detritivores and predators in forest ecosystems.

### Environmental factors

The abiotic factors we measured only had a small influence on the functional composition of detritivores and predators (Tables 3 and 5). A correlation between abiotic factors and feeding traits can reveal a correlation between the feeding traits and unmeasured traits related to abiotic factors or dispersion (Gravel et al. 2016). If abiotic or landscape factors have a strong filtering role, it can create trait mismatches between consumers and their resources (Le Provost et al. 2017). For example, bees in recently burned forests were shown to have shorter tongues than bees in unburned sites; this covariation has perhaps nothing to do with the interaction with available flowers, rather it is probably due to a correlation between tongue length and nesting sites, which are strongly affected by fire (Moretti et al. 2009). We observed at the Mont Écho site that trait matches between detritivores and leaf litter correspond to our top‐down control hypotheses, in contrast to the other two sites. Mont Écho differed from other sites by having a higher variability in soil humidity (between 4.5–37.7%) and a higher proportion of coniferous litter (22% compared to <1% in other sites). Humidity level is an important filter for Diplopoda and Isopoda (David and Handa 2010) and could be particularly important in coniferous forests (Wytwer and Tracz 2003). Humidity variation at the Mont Écho site may thus have driven the negative covariation between traits. Also, we included in the analyses all leaf litter species, although, we are not sure that the detritivore species found at Mont Écho actually consumed coniferous needle litter. Litters of *A. balsamea* and *Picea* sp. are the thickest and toughest species across all sites (Appendix S3; Table S1) and could be unpalatable even for the strongest detritivore with the largest mandibular gape in this site. Unmeasured traits such as chemical defense compounds could also prevent or reduce interactions (Cárcamo et al. 2000).

The relative importance of environmental factors to resource community structure varied as a function of consumer functional or taxonomic structure (Tables 3 and 5). This is particularly clear for detritivores: temperature is the dominant factor for taxonomic structure while litter community structure (principally the third axis) is the dominant factor for functional structure. Such results show that environmental factors that impact taxonomic community structure may not have similar effects on the functional structure of feeding traits. It is worth noting, however, that we did not include sites with extreme environmental conditions. In addition, resource community structure was integrated in the analysis as the score of the first three PCA axes; these axes are a surrogate of the community structure but do not reflect it perfectly, which may have influenced our results.

## Conclusion

Our study shows that the traits of interacting trophic levels (litter–detritivores and predator–prey) covary in space. Interestingly, similar results were observed in a different ecosystem between plants and herbivores with equivalent traits (leaf toughness–biting force; Le Provost et al. 2017). While these results help to understand assembly processes across trophic levels, they are mainly based on bottom‐up dynamics. However, top‐down control dynamics are also known to occur in the soil ecosystem (Buchkowski 2016). Although we observed trait covariation at the Mont Écho site for detritivores and forest litter that corresponded to the hypothesized effect of top‐down control, other factors may also be at play. Observing top‐down control could be particularly hard to detect in species‐rich ecosystems that contain many indirect interspecific interactions (Cazelles et al. 2016). A potential way to consider top‐down effects could be by looking at the distribution of antipredator traits. Stronger predation pressure on a particular point of prey functional space could favor the presence of antipredator traits in the species sharing these traits values. Nonetheless, increasing our understanding of top‐down processes is required to better understand food web dynamics (Buchkowski 2016) and to understand the impact of higher trophic levels on ecosystem functioning (Wang and Brose 2018).
