# Supplementary material for: Traits of litter‐dwelling forest arthropod predators and detritivores covary spatially with traits of their resources
Source: Ecology. 2019 Aug 14;100(10):e02815. doi: 10.1002/ecy.2815 (PMC6852231; doi:10.1002/ecy.2815)
Supplement: Supplementary file 1 [file ECY-100-na-s001.pdf]

Supporting Information. Brousseau, P.-M., Gravel, D. and Handa, I.T. 2019. Traits of litter-dwelling forest arthropod predators and detritivores covary spatially with traits of their resources. *Ecology.*

Appendix S1 - Trait measurement

Body volume

Body volume was measured for all macroarthropods and consists of three measures: body length, body width and body height. We measured the body length as the distance from the front of the head to the apex of the abdomen in dorsal view (Figs. S1 and S2). For prognathous arthropods, the frontal end was the anterior margin of the clypeus; for hypognathous arthropods, the frontal end was the anterior margin of the front. Body width represented the largest part of the thorax (excluding appendices or hairs) in dorsal view (Figs. S1 and S2). Body height represented the highest point (around mid-section) of the thorax in lateral view. For spiders, body width and height were measured on the cephalothorax. Height was always measured behind the eye so to avoid modified eye platform of some males (Fig. S3). Body volume was calculated so as to best approximate the body shape of each taxon (Table S1).

Table S1. Equation used to determine the body volume of arthropods based on the approximated shape of the taxon where a = width/2, b = height/2, c = length.

Body shape	Equation	Taxa
Ellipsal cylinder	$\pi abc / 4$	Diplopoda, Larvae
Half ellipsoid	$2\pi abc / 3$	Araneae, Isopoda, Opiliones
Ellipsoid	$4\pi abc / 3$	Carabidae

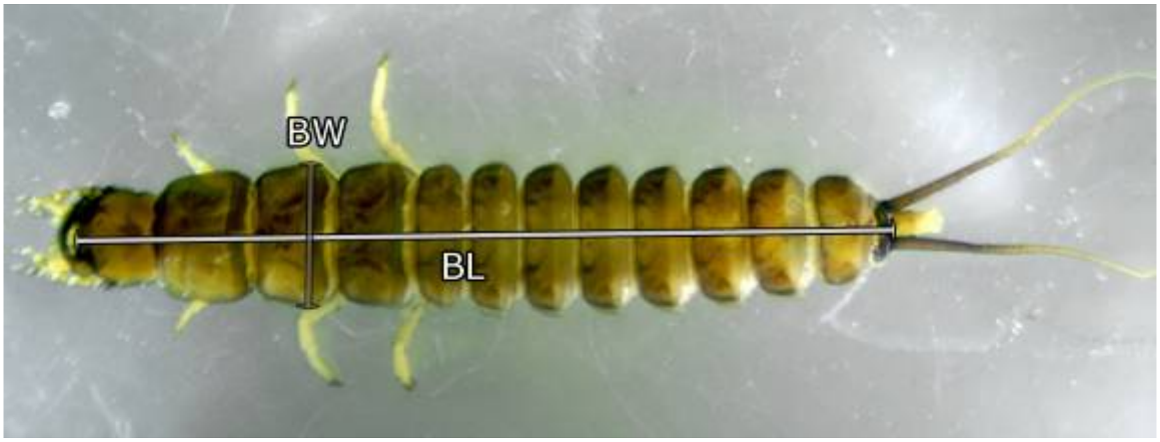

Figure S1 Measurement of body length (BL) and body width (BW) on mandibulated arthropods. Specimen = *Chlaenius* sp. (larva).

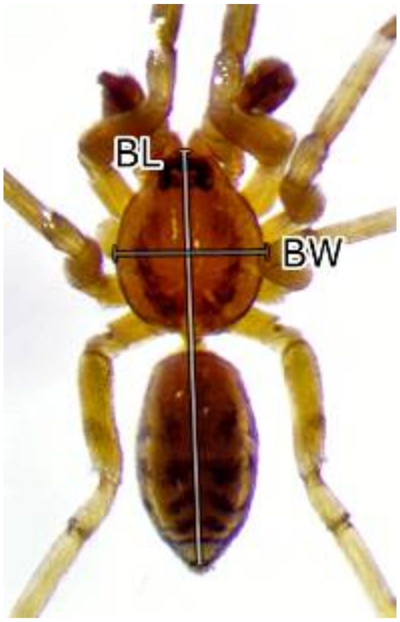

Figure S2 Measurement of body length (BL) and body width (BW) on spiders. Specimen = *Phrurotimpus alarius*.

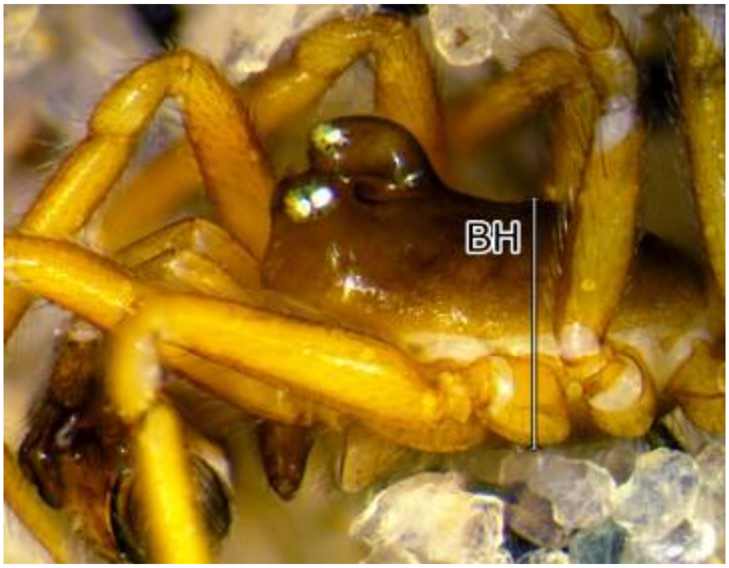

Figure S3 Measurement of body height (BH) on spiders. Specimen = *Walckenaeria castanea* ♂.

Cuticular toughness

Cuticular toughness was measured with a Pesola® Medio-Line pressure set to which we added an entomological pin of size 2 (diameter = 0.45 mm). Toughness was the pressure required to break through the integument with the pin in g mm^{-2}. A value of zero was given for very small and soft prey, while a value of 1 was given to small prey with sclerotized cuticle (e.g. small Coleoptera larvae) (Fig. S4). Toughness of the cuticle can be variable depending on the body part, so measurements were consistently taken at the level of the abdomen from the dorsal side.

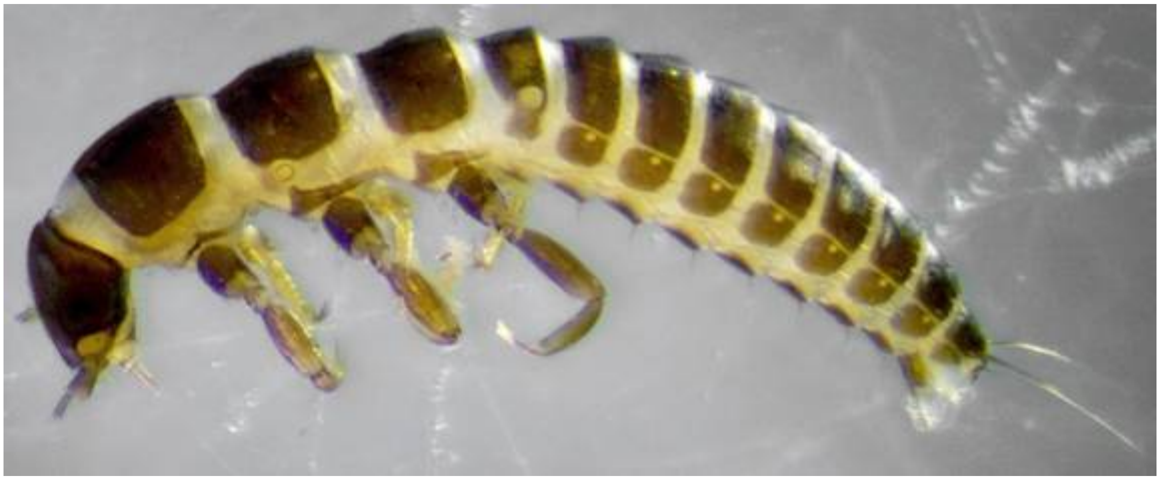

Figure S4 Example of small Coleoptera larva with sclerotized cuticle. Specimen = *Meloe* sp.

Biting force

Biting force at mouthpart tip was determined with the formula $h \times b/c$. For Carabidae (Figs. S5 and S6) and Diplopoda (Fig. S7), h was the width of the head behind the eyes (i.e. the attachment point of the adductor muscle of the mandibles), b was the basal width of the mandible between the upper condyle and the insertion point of the adductor muscle, and c its length from the upper condyle to the tip (Wheater and Evans 1989; Clissord 2007; Brousseau et al., 2018). Temples were included in the measurement when developed (Fig. S6). Measurements where made on the left mandible.

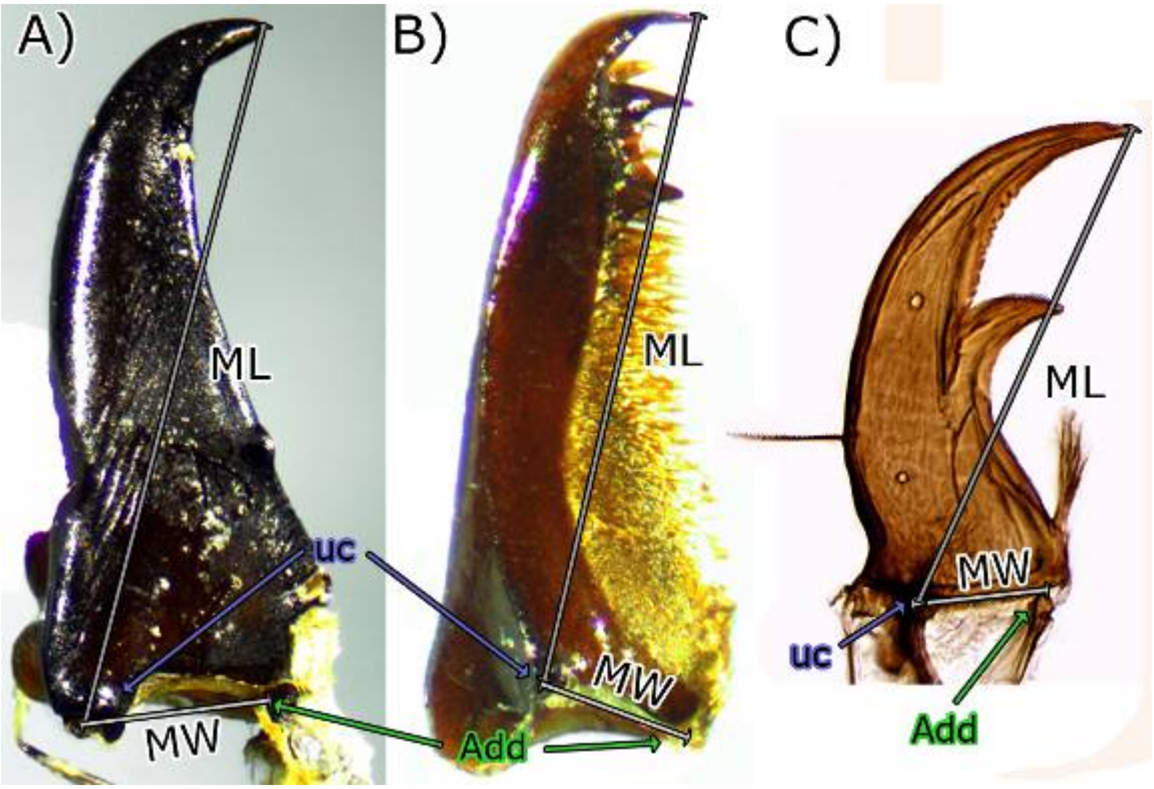

Figure S5 Measurement of the basal width of mandible (MW) and mandibular length (ML) on three types of Carabidae mandibles. A) *Pterostichus rostratus*; B) *Sphaeroderus lecontei*; C) *Platynus* sp. (larva). Add = insertion point of the adductor muscle; uc = upper condyle.

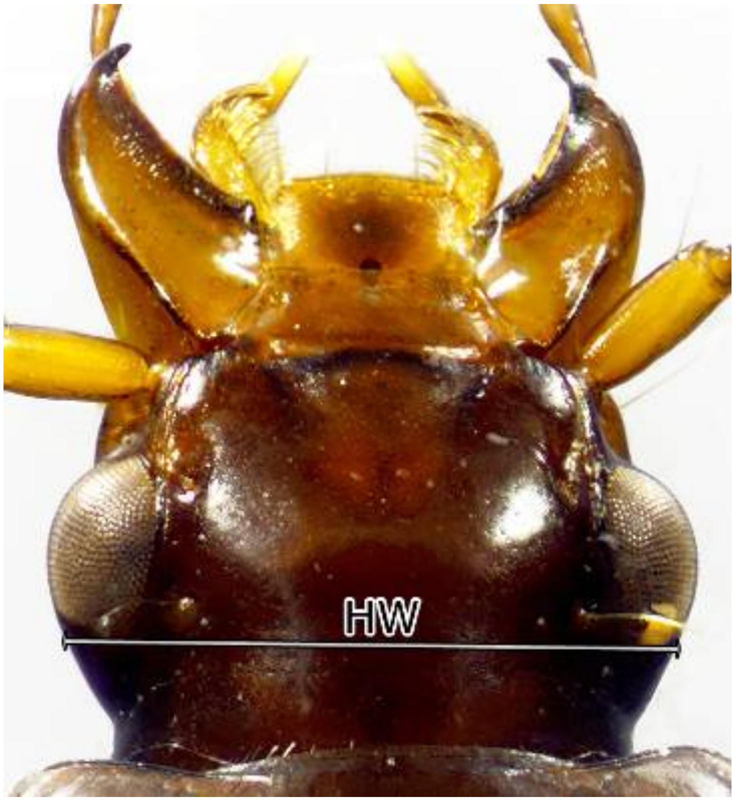

Figure S6 Measurement of head width (HW) of Carabidae. Specimen = *Synuchus impunctatus*.

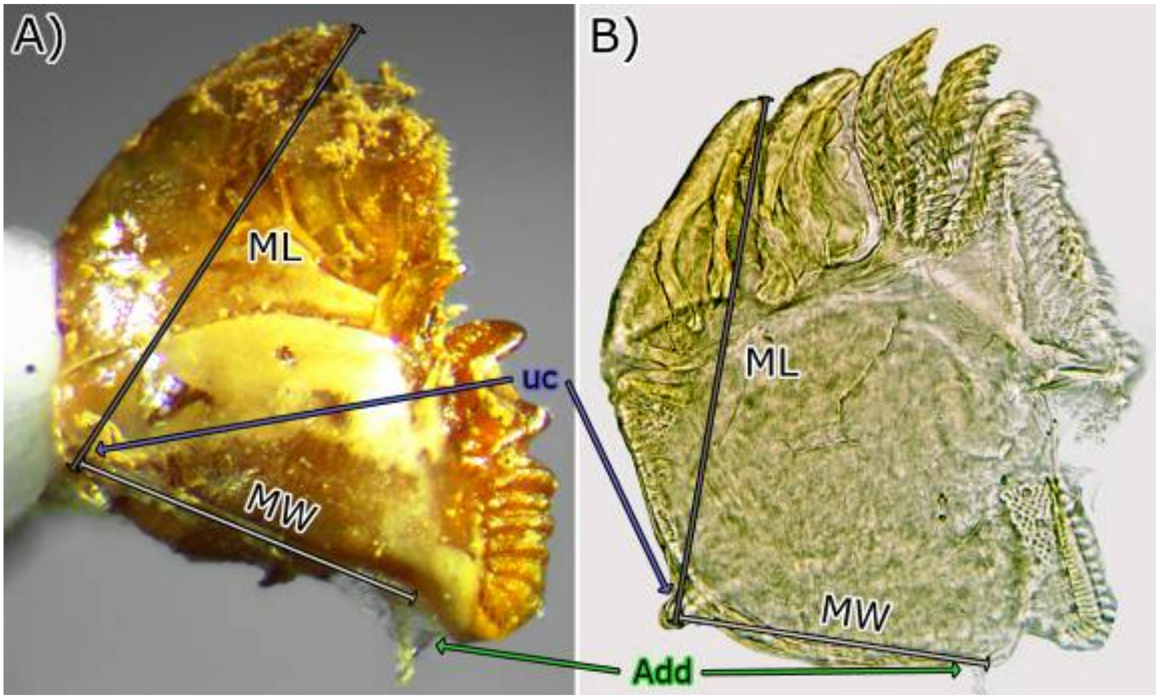

Figure S7 Measurement of the basal width of mandible (MW) and mandibular length (ML) on the mandibles of two Diplopoda species. A) *Narceus americanus*; B) *Nopoiulus kochii*. Add = insertion point of the adductor muscle; uc = upper condyle.

For arachnids (Fig. 8S), *h* is the size (length x width) of the chelicerae measured in frontal view, *b* is the basal width of the movable digit and *c* its length from the condyle to the tip (without considering the curvature of the digit) (van der Meijden, Herrel & Summers 2010; van der Meijden et al. 2012). The length of the chelicerae was always measured in the mid section, while width was the largest point in the basal half of the chelicera. All measurements were done on the left chelicera.

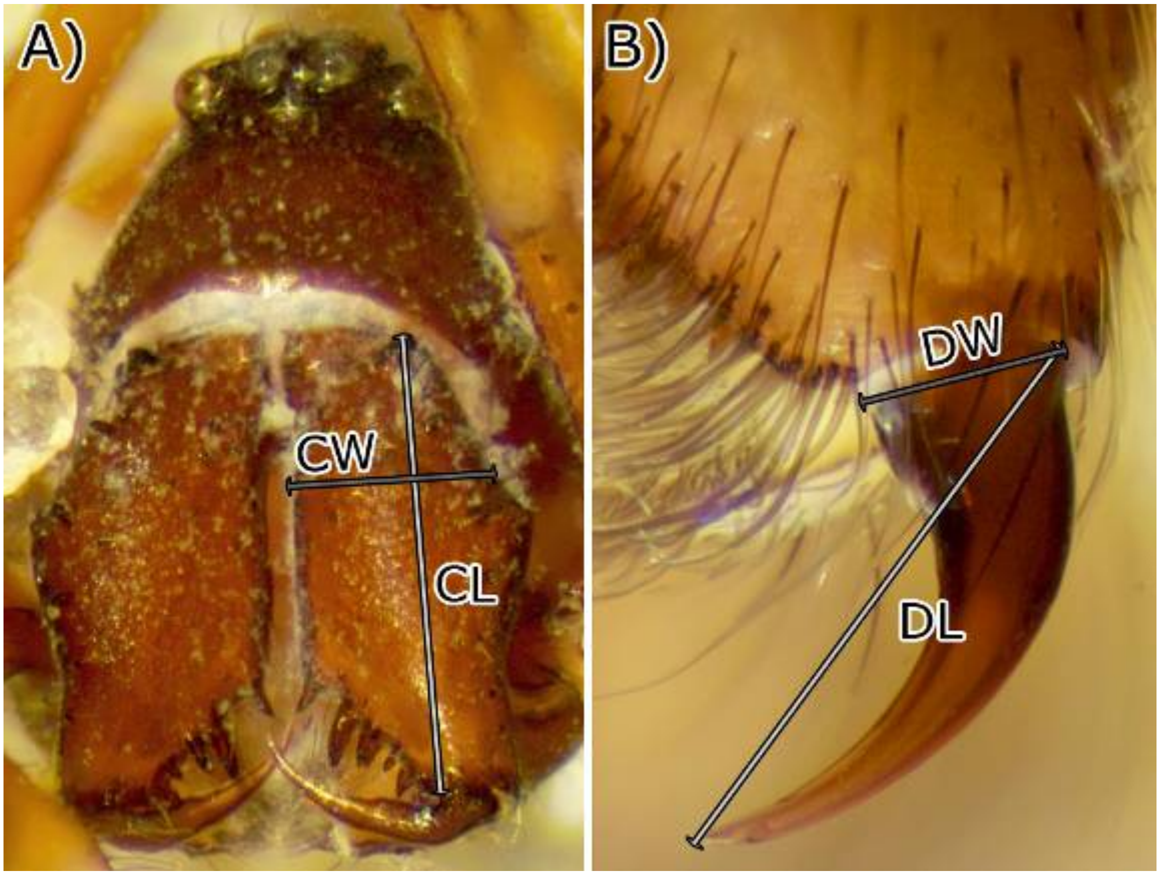

Figure S8 Measurement of the A) chelicerae length (CL) and width (CW) and B) digit length (DL) and width (DW) of arachnids. A) *Erigone atra* ♂; B) *Agelenopsis potteri*.

Molar plate force

For Diplopoda, force of the molar plate was determined with the formula $h \times b/c$ where *h* was the longest length of the stipe (i.e. the attachment point of the abductor muscle of the mandible) in lateral view (Fig. S9), *b* was the distance between the condyle and the insertion point of the abductor muscle, and *c* was the distance between the condyle and the external mid-point of the molar plate (Fig. S10). In some species, the stipe of males is expanded ventrally, but the muscles do not attach in these extensions; thus the extensions were not considered in the measurement of stipe length (Fig. S9). The distance between the condyle and the insertion point of the abductor muscle was easier to measure from the rear of the mandible (Fig. S10B). All measurements were done on the left mandible.

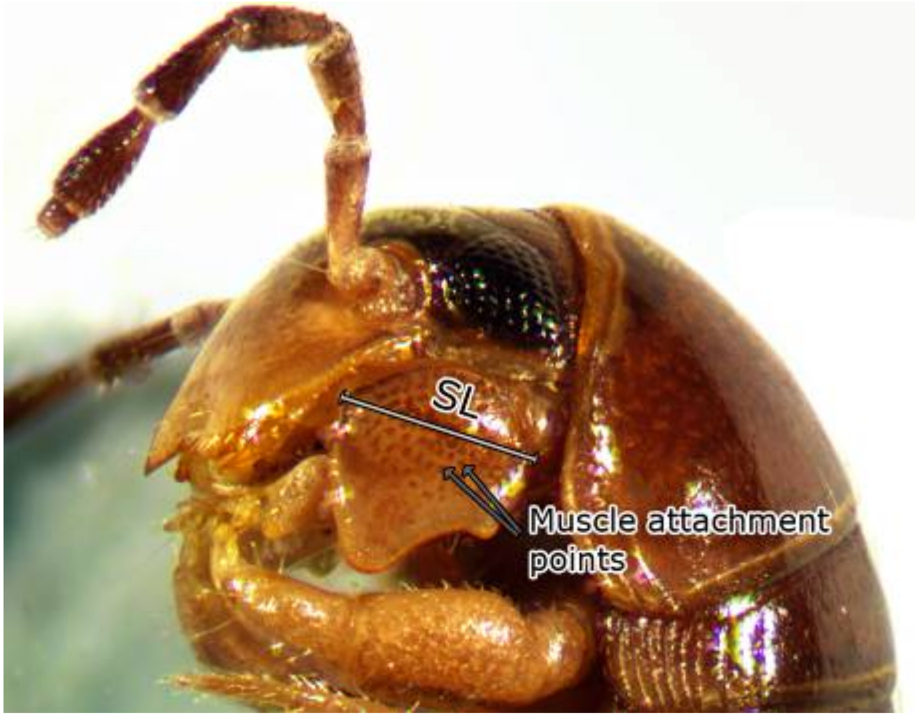

Figure S9 Measurement of the stipe length (SL) on Diplopoda. Specimen = *Uroblaniulus canadensis* ♂.

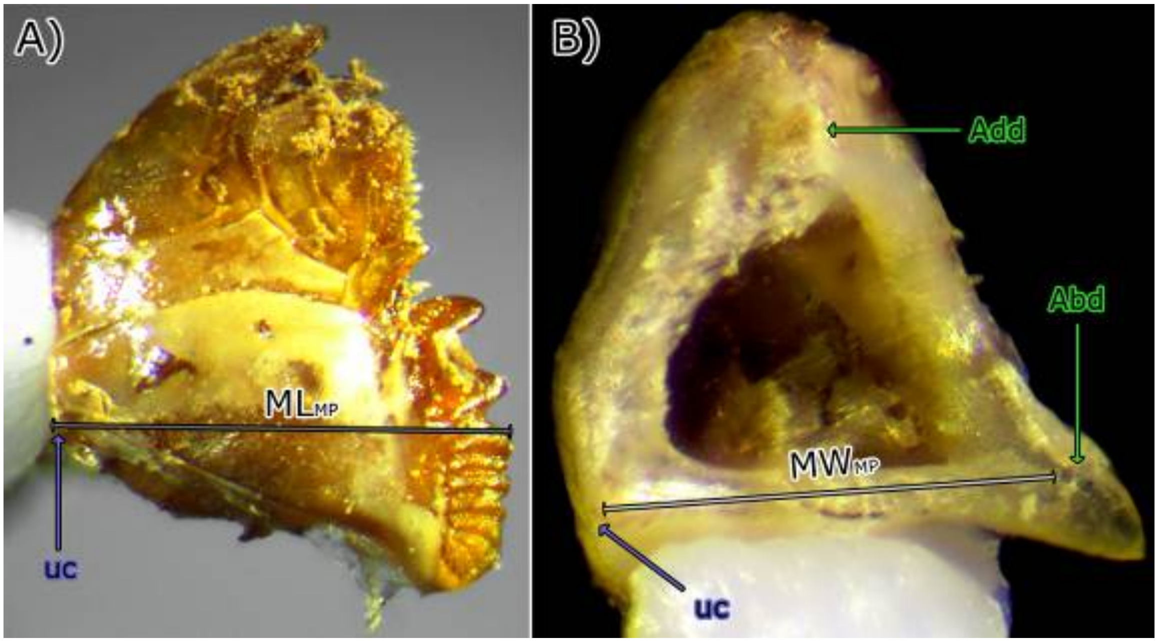

Figure S10 Measurement of the basal width of mandible (MW_{MP}) and mandibular length from the molar plate (ML_{MP}) on the mandibles of Diplopoda. A) Dorsal view; specimen = *Narceus americanus*. B) Rear view; specimen = *Cylindroiulus caeruleocinctus*. Abd = insertion point of the abductor muscle; Add = insertion point of the adductor muscle; uc = upper condyle.

Mechanical advantage

The mechanical advantage is the width/length ratio of the mouthparts. For Carabidae and Diplopoda, the width of the mandible was measured between the upper condyle and the attachment point of the adductor muscle, and the length from the upper condyle to mandibular tip (Fig. S5, S7). For arachnids, the width of the movable digit of the chelicera was measured from the condyle to the attachment point of the adductor muscle, and the length from the condyle to the tip of the digit (without considering the curvature of the digit) (Fig. S8).

Mandibular gape

The mandibular gape represents the distance between both mandibles when fully open (Fig. S11a). Most of the time, it was impossible to kept mandibles open while measuring. In these cases, one mandible was fully opened and the labial seta nearer to the mandible tip was identified. The mandibular gape was then measured as the distance between the identified labial seta on both sides; e.g. the fourth labial seta was used for Narceus americanus (Fig. S11b).

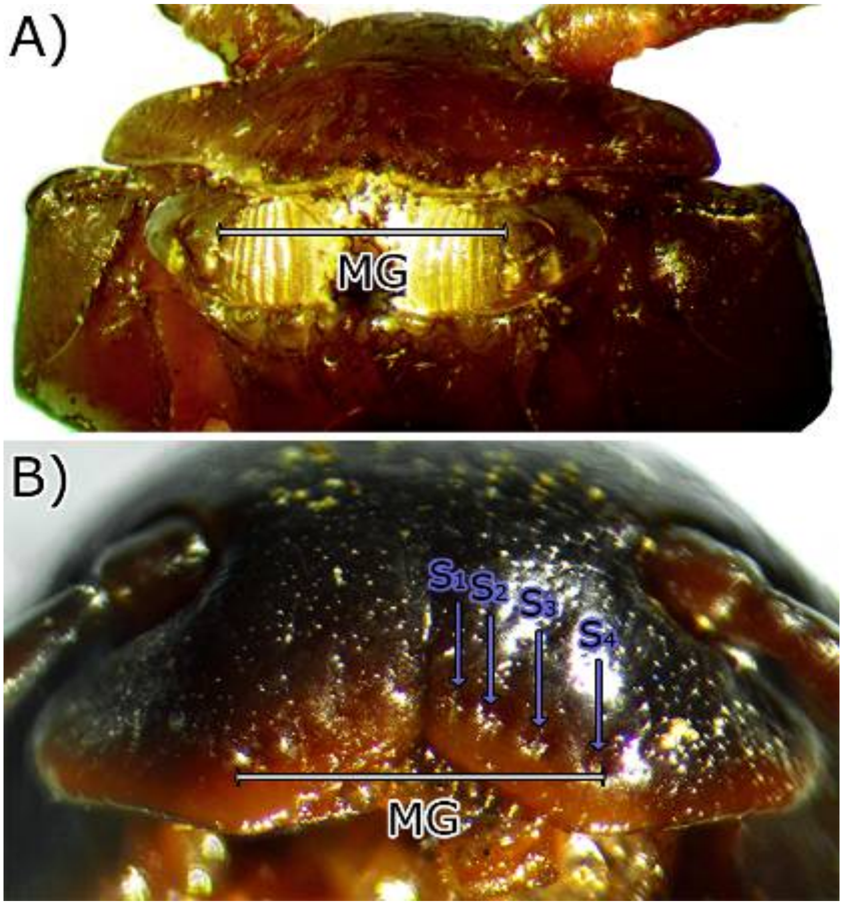

Figure S11 Measurement of the mandibular gape through A) a direct measure or B) an indirect measure. A) *Pseudopolydesmus serratus*; B) *Narceus americanus*. S1-S4 = Labial seta 1 to seta 4

References

Brousseau, P.-M., D. Gravel, and I. T. Handa. 2018b. Trait-matching and phylogeny as predictors of predator-prey interactions involving ground beetles. Functional Ecology 32:192-202.

Clissold, F.J. 2007. The biomechanics of chewing and plant fracture: mechanisms and implications. Advances in insect physiology 34:317-372.

van der Meijden, A., A. Herrel, and A. Summers. 2010. Comparison of chela size and pincer force in scorpions; getting a first grip. Journal of Zoology 280:319-325.

van der Meijden, A., F. Langer, R. Boistel, P. Vagovic, and M. Heethoff 2012. Functional morphology and bite performance of raptorial chelicerae of camel spiders (Solifugae). The Journal of experimental biology 215:3411-3418.

Wheater, C. P., and M. E. G. Evans. 1989. The mandibular forces and pressures of some predacious Coleoptera. Journal of Insect Physiology 35:815-820.
