# Supplementary material for: Traits of litter‐dwelling forest arthropod predators and detritivores covary spatially with traits of their resources
Source: Ecology. 2019 Aug 14;100(10):e02815. doi: 10.1002/ecy.2815 (PMC6852231; doi:10.1002/ecy.2815)
Supplement: Supplementary file 2 [file ECY-100-na-s002.pdf]

Supporting Information. Brousseau, P.-M., Gravel, D. and Handa, I.T. 2019. Traits of litter-dwelling forest arthropod predators and detritivores covary spatially with traits of their resources. *Ecology.*

Appendix S2 - Supplementary graphics

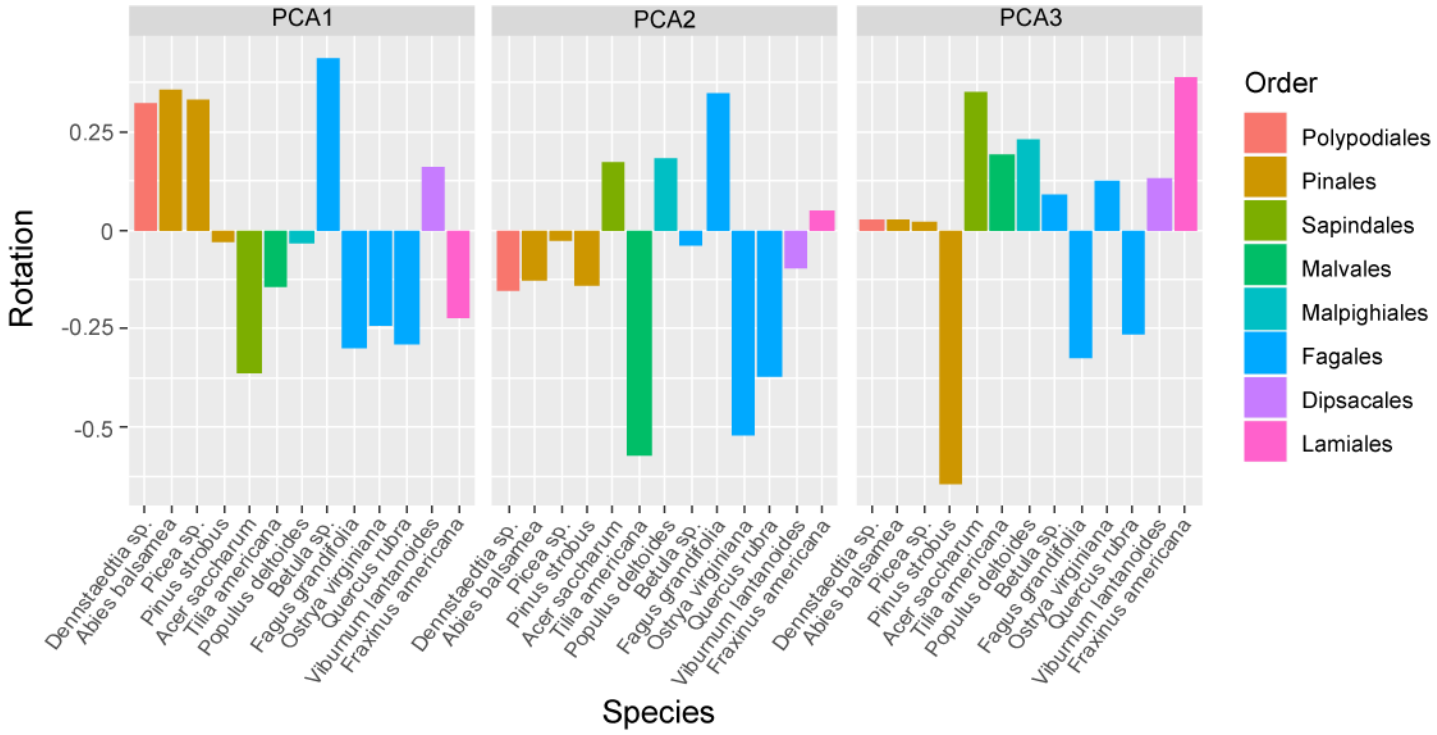

Figure S1 Leaf litter species rotation on the three first axes of a PCA on the abundance of leaf litter in three forested sites in southern Québec.

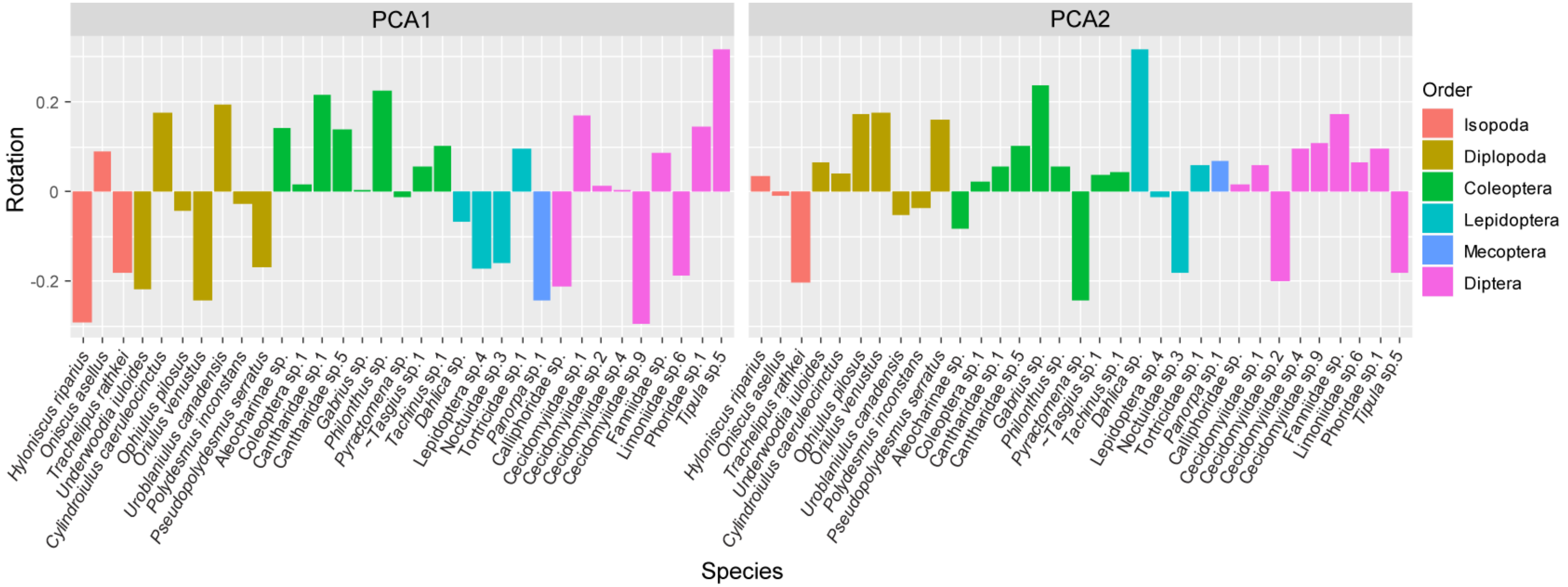

Figure S2 Prey species rotation on the two first axes of a PCA on the abundance of leaf litter in three forested sites in southern Québec. Only the species with a rotation score > 0.15 on one of the first 3 axes of the PCA (33 out of 135) are represented.

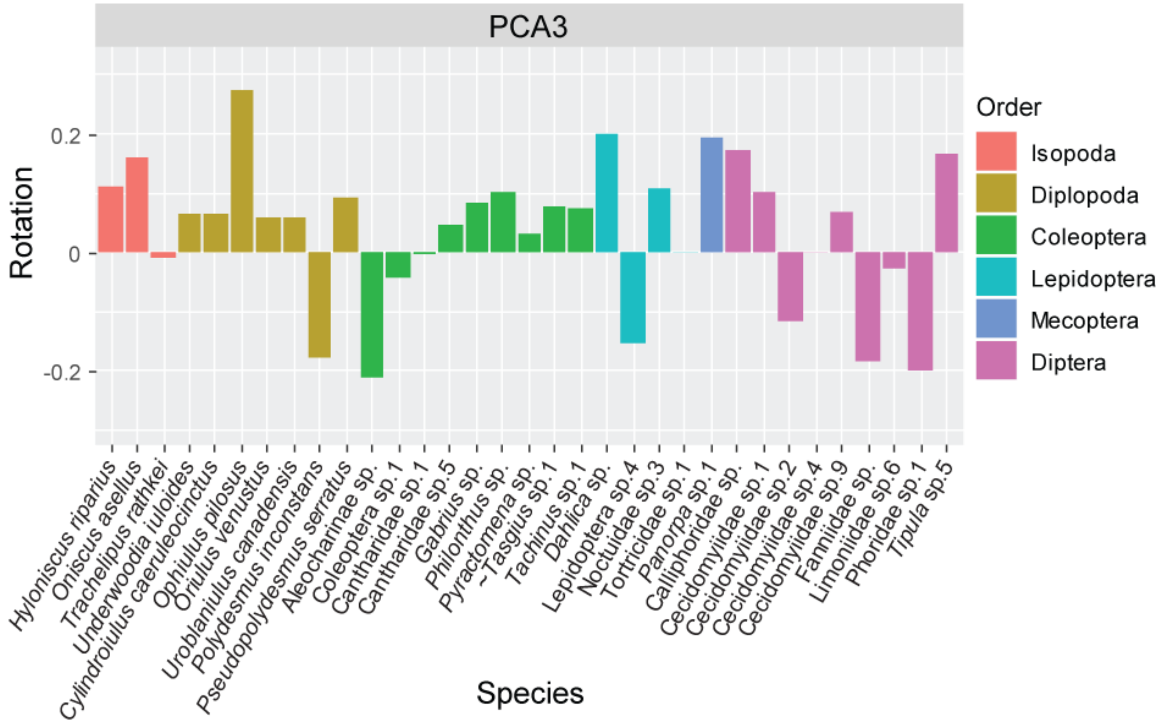

Figure S3 Prey species rotation on the third axis of a PCA on the abundance of leaf litter in three forested sites in southern Québec. Only the species with a rotation score > 0.15 on one of the first 3 axes of the PCA (33 out of 135) are represented.

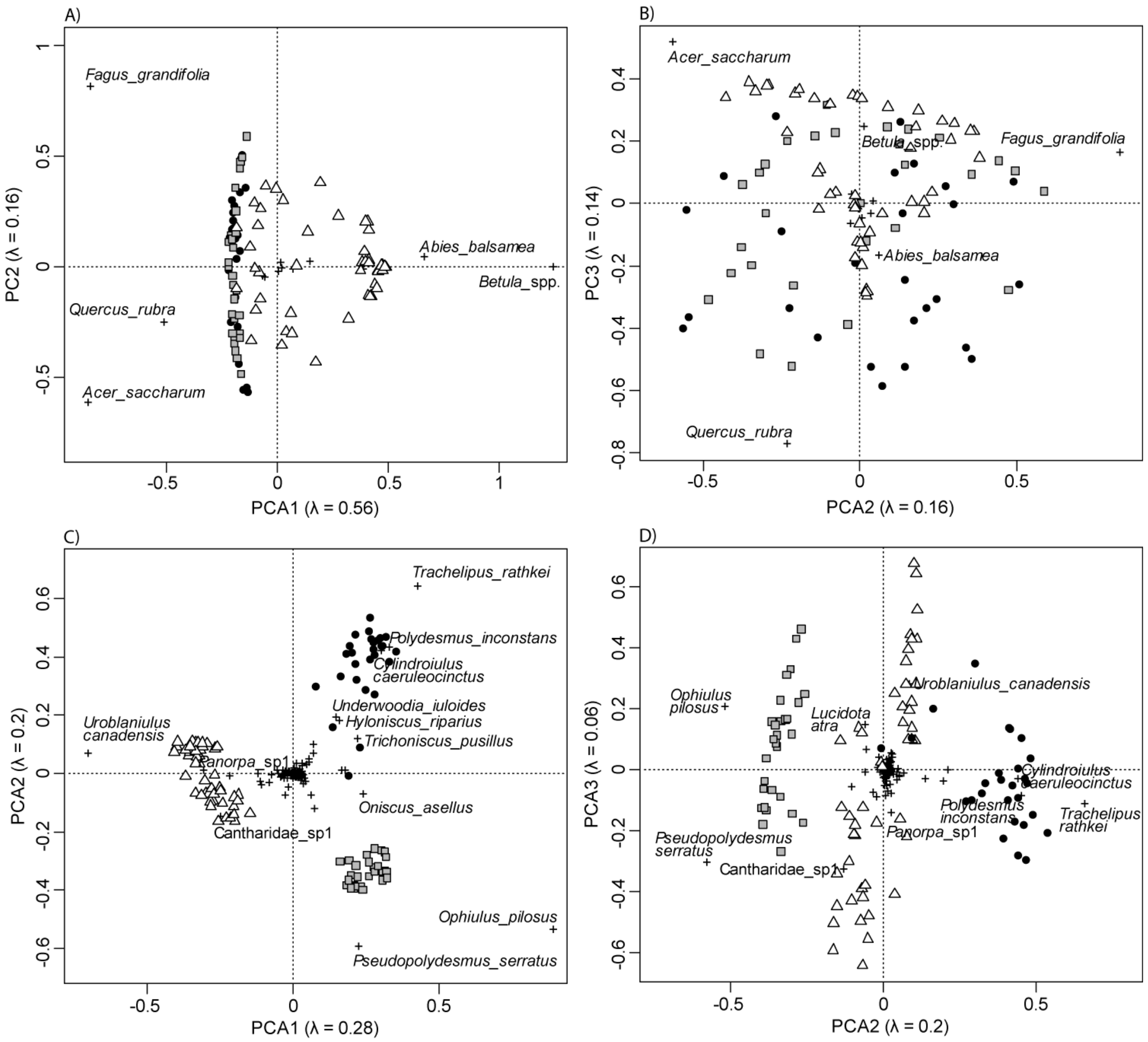

Figure S4 Principal component analysis (PCA) on the abundance of A-B) leaf litter C-D) and prey species found in three forested sites in southern Québec. A-C) PCA axes 1 and 2; B-D) axes 2 and 3. Circle = Mont-St-Bruno; square = Mont-St-Hilaire; triangle = Mont-Écho; + = species. For both leaf litter (R^2=0.45, P<0.001) and prey (R^2=0.46, P<0.001), the sites are significantly different based on Adonis (PERMANOVA) analysis on Bray-Curtis dissimilarity.
