# Supplementary material for: Traits of litter‐dwelling forest arthropod predators and detritivores covary spatially with traits of their resources
Source: Ecology. 2019 Aug 14;100(10):e02815. doi: 10.1002/ecy.2815 (PMC6852231; doi:10.1002/ecy.2815)
Supplement: Supplementary file 3 [file ECY-100-na-s003.pdf]

Supporting Information. Brousseau, P.-M., Gravel, D. and Handa, I.T. 2019. Traits of litter-dwelling forest arthropod predators and detritivores covary spatially with traits of their resources. *Ecology.*

Appendix S3 - Tables of abundance and functional trait values

Table S1 Total mass and range of functional trait values (± standard error) of partially decomposed leaf litter collected on the groundof three forests of southern Québec, Canada. SB = Mont-St-Bruno, SH = Mont-St-Hilaire, MÉ = Mont-Écho.

	Total mass (g)			Functional trait value		
**Species**	**SB**	**SH**	**MÉ**	**Toughness ($g\ mm^{-2}$)**	**Tickness (mm)**	**LDMC (%)**
Abies balsamea (L.) Miller	0	0	146.39	76 ± 16	0.38 ± 0.09	56.7 ± 13.4
Acer saccharum Marshall	247.74	365.95	155.69	29 ± 13	0.14 ± 0.06	43.1 ± 16.4
Betula spp.	0	0	221.92	12 ± 12	0.17 ± 0.05	34.8 ± 16.7
Dennstaedtia punctilobula (Michaux) Moore	0	0	35.99	14 ± 4	0.09 ± 0.02	56.8 ± 6.1
Fagus grandifolia Ehrhart	383.49	324.6	116.64	36 ± 25	0.13 ± 0.05	46.6 ± 20.2
Fraxinus americana L.	4.6	7.96	0.1	21 ± 12	0.18 ± 0.03	30.5 ± 7
Ostrya virginiana (Miller) Koch	3.67	7.59	0	15 ± 12	0.14 ± 0.03	60.9 ± 15.1
Picea sp.	0	0	5.75	117 ± 43	0.64 ± 0.11	49.5 ± 12.8
Pinus strobus L.	4.18	0	0.03	65 ± 17	0.28 ± 0.03	54.2 ± 5.2
Populus deltoides Batram	0.1	0	0	24 ± 11	0.23 ± 0	91.7 ± 0
Quercus rubra L.	235.57	126.87	0	74 ± 31	0.21 ± 0.05	56.5 ± 15.1
Tilia americana L.	1.66	0.53	0	96 ± 25	0.2 ± 0.03	56.8 ± 0
Viburnum lantanoides Michaux	0	0	1.58	11 ± 8	0.1 ± 0.02	62.7 ± 19.1

Table S2 Abundance and range of functional trait values (± standard error) of adult (A) and larval (L) Carabidae caught in pitfall traps in three forests of southern Québec, Canada. Details on trait measurements are provided in Appendix S1. St. = Development stage, SB = Mont-St-Bruno, SH = Mont-St-Hilaire, MÉ = Mont-Écho.

		Abundance			Functional trait value		
**Species**	**St.**	**SB**	**SH**	**MÉ**	**Body volume (mm^3)**	**Biting force index**	**Mechanical advantage**
Agonum fidele Casey	A	0	3	3	23.5 ± 4.2	0.33 ± 0.04	0.26 ± 0.03
Agonum melanarium Dejean	A	1	0	0	33.6	0.52	0.4
Agonum retractum LeConte	A	1	1	714	11.5 ± 0.4	0.29 ± 0.05	0.28 ± 0.05
Agonum sp.	L	0	2	18	5.4 ± 0.9	0.29 ± 0.02	0.33 ± 0.03
Amphasia interstitialis (Say)	A	1	1	1	52.1 ± 7.3	0.68 ± 0.04	0.35 ± 0.04
Bembidion mimus Hayward	A	0	0	1	1.3	0.22	0.76
Bembidion wingatei Bland	A	0	0	4	4.1 ± 0.3	0.23	0.27 ± 0.01
Calathus gregarius (Say)	A	0	0	370	22.1 ± 2.8	0.4 ± 0.03	0.32 ± 0.03
Calathus ingratus Dejean	A	0	0	196	23.6 ± 3.7	0.37 ± 0.03	0.29 ± 0.02
Calosoma frigidum Kirby	A	1	4	2	572.5 ± 88.1	0.84 ± 0.11	0.26 ± 0.03
Calosoma frigidum Kirby	L	0	2	0	41.6 ± 34.9	1.07 ± 0.4	0.57 ± 0.1
Carabus chamissonis von Waldheim	L	1	0	0	373.2	0.84	0.29
Carabus maeander Fischer	A	0	0	1	328.3	0.73	0.32
Carabus nemoralis Müller	A	728	0	0	678.8 ± 89.3	0.78 ± 0.12	0.22 ± 0.03
Carabus nemoralis Müller	L	32	0	0	146.3 ± 127.6	0.64 ± 0.17	0.28 ± 0.02
Chlaenius emarginatus Say	A	65	3	0	77.7 ± 13	0.43 ± 0.05	0.21 ± 0.02
Chlaenius sp.	L	3	0	0	17.7 ± 10.5	0.43 ± 0.08	0.34 ± 0.02
Cymindis cribricollis Dejean	A	1	50	1	28.4 ± 1.5	0.38 ± 0.04	0.24 ± 0.02
Cymindis limbata Dejean	A	0	1	0	19.4	0.46	0.33
Cymindis unicolor Kirby	A	0	1	0	11.5	0.34	0.28
Cymindis sp.	L	2	4	0	6.5 ± 4.2	0.29 ± 0.08	0.34 ± 0.03
Dicaelus politus Dejean	A	0	8	0	78 ± 5.9	0.8 ± 0.05	0.37 ± 0.02
Dicaelus politus Dejean	L	0	1	0	35	0.26	0.28
Elaphrus fuliginosus Say	A	0	0	1	32.8	0.32	0.21
Gastrellarius honestus (Say)	A	0	0	7	24.1 ± 1.3	0.36 ± 0.01	0.28 ± 0.02
Harpalus faunus Say	A	0	0	1	83.4	1.07	0.41
Harpalus somnulentus Dejean	A	0	1	0	34.9	0.58	0.28
Loricera pilicornis (Fabricius)	A	0	0	1	15.7	0.38	0.36
Myas cyanescens Dejean	A	71	64	0	106.6 ± 47.8	0.6 ± 0.06	0.24 ± 0.01
Myas cyanescens Dejean	L	1	3	0	58.1 ± 19.4	0.56 ± 0.09	0.35 ± 0.04
Notiophilus aeneus (Herbst)	A	1	1	9	18.2 ± 0.4	0.45 ± 0.09	0.23 ± 0.08
Notiophilus aeneus (Herbst)	L	0	0	2	1.4 ± 0.7	0.18 ± 0.04	0.25 ± 0.02
Olisthopus parmatus Say	A	2	6	0	14.9 ± 1	0.36 ± 0.02	0.32 ± 0.02
Patrobus longicornis Say	A	0	3	0	58.1 ± 12.1	0.53 ± 0.03	0.28 ± 0.01
Platynus mannerheimii Dejean	A	0	0	6	40.5 ± 6.1	0.47 ± 0.06	0.29 ± 0.03
Platynus opaculus (LeConte)	A	8	19	169	44.8 ± 5.8	0.5 ± 0.04	0.29 ± 0.03
Platynus tenuicollis (LeConte)	A	0	0	2	25.2 ± 2.3	0.32	0.26
Platynus sp.	L	0	0	11	0.8 ± 0.4	0.16 ± 0.02	0.34 ± 0.04
Poecilus lucublandus (Say)	A	5	0	0	69.9 ± 6.7	0.57 ± 0.07	0.29 ± 0.02
Pterostichus adoxus (Say)	A	15	32	32	82.8 ± 13.9	0.65 ± 0.04	0.28 ± 0.01
Pterostichus adstrictus Eschscholtz	A	0	0	707	50 ± 5.6	0.55 ± 0.04	0.28 ± 0.02
Pterostichus adstrictus Eschscholtz	L	0	0	16	24.4 ± 4.8	0.52 ± 0.06	0.3 ± 0.03
Pterostichus coracinus (Newman)	A	832	307	959	123.1 ± 37.7	0.87 ± 0.06	0.3 ± 0.02
Pterostichus coracinus (Newman)	L	0	0	1	50.9	0.45	0.23
Pterostichus diligendus (Chaudoir)	A	0	0	36	63.4 ± 6.4	0.56 ± 0.06	0.27 ± 0.03
Pterostichus diligendus (Chaudoir)	L	0	0	1	17.3	0.32	0.23
Pterostichus lachrymosus Newman	A	937	220	1	167.5 ± 32.4	0.87 ± 0.03	0.29 ± 0.02
Pterostichus lachrymosus Newman	L	3	0	0	29.8 ± 9.3	0.37 ± 0.04	0.23 ± 0.03

Pterostichus melanarius Illiger	A	246	14	0	148.9 ± 23.5	0.81 ± 0.04	0.28 ± 0.01
Pterostichus mutus Say	A	9	12	1	57.3 ± 12.1	0.58 ± 0.06	0.29 ± 0.02
Pterostichus pensylvanicus LeConte	A	228	205	0	64.6 ± 11.9	0.66 ± 0.04	0.3 ± 0.02
Pterostichus pensylvanicus LeConte	L	1	0	0	23.3	0.54	0.34
Pterostichus rostratus (Newman)	A	0	60	17	115.9 ± 14.7	0.8 ± 0.14	0.23 ± 0.04
Pterostichus rostratus (Newman)	L	0	4	1	29.1 ± 6.6	0.6 ± 0.11	0.36 ± 0.07
Pterostichus tristis (Dejean)	A	14	45	95	60 ± 3	0.59 ± 0.04	0.28 ± 0.01
Scaphinotus viduus (Dejean)	A	0	0	50	936.9 ± 135.7	0.4 ± 0.1	0.14 ± 0.03
Scaphinotus viduus (Dejean)	L	0	0	1	107.4	1.34	0.77
Sphaeroderus canadensis Chaudoir	A	0	26	111	90.2 ± 10.6	0.59 ± 0.79	0.43 ± 0.56
Sphaeroderus canadensis Chaudoir	L	0	1	14	39.1 ± 26.9	0.34 ± 0.04	0.3 ± 0.05
Sphaeroderus lecontei Dejean	A	6	67	100	50.4 ± 5.2	0.31 ± 0.04	0.21 ± 0.01
Sphaeroderus lecontei Dejean	L	1	13	4	31.9 ± 22.4	0.33 ± 0.06	0.3 ± 0.07
Synuchus impunctatus (Say)	A	1916	3036	1563	29.4 ± 6.9	0.44 ± 0.07	0.29 ± 0.03
Trechus apicalis Motschulsky	A	0	0	43	2.6 ± 0.2	0.23 ± 0.03	0.32 ± 0.04

Table S3 Abundance and range of functional trait values (± standard error) of spiders (Araneae) and Opiliones caught in pitfall traps in three forests of southern Québec, Canada. Details on trait measurements are provided in Appendix S1. SB = Mont-St-Bruno, SH = Mont-St-Hilaire, MÉ = Mont-Écho.

		Abundance			Functional trait value		
**Family**	**Species**	**SB**	**SH**	**MÉ**	**Body volume (mm^3)**	**Biting force index**	**Mechanical advantage**
**Araneae**							
Agelenidae	*Agelenopsis potteri* (Blackwall)	6	3	0	16.7 ± 2.4	0.51 ± 0.05	0.31 ± 0.01
	Agelenopsis utahana (Chamberlin & Ivie)	12	37	8	14 ± 2.6	0.43 ± 0.06	0.29 ± 0.01
	Coras juvenilis (Keyserling)	6	9	0	14.4 ± 5.1	0.4 ± 0.01	0.25 ± 0.03
	Coras montanus (Emerton)	0	0	1	16.3 ± 1.7	0.55	0.28
	Wadotes calcaratus (Keyserling)	1	52	824	10.2 ± 2.1	0.45 ± 0.11	0.34 ± 0.11
	Wadotes hybridus (Emerton)	331	333	25	28.3 ± 6.1	0.6 ± 0.11	0.23 ± 0.05
Amaurobiidae	*Amaurobius borealis* Emerton	0	1	79	2.4 ± 0.4	0.28 ± 0.05	0.33 ± 0.02
	Callobius bennetti (Blackwall)	3	8	46	17.7 ± 6.5	0.64 ± 0.12	0.34 ± 0.05
Clubionidae	*Clubiona bishopi* Edwards	0	0	19	1.6 ± 0.6	0.23 ± 0.06	0.38
	Clubiona canadensis Emerton	0	2	8	8.5 ± 2.7	0.36	0.31 ± 0.02
	Clubiona obsesa Hentz	1	0	0	11	0.55	0.39
	Clubiona spiralis Emerton	21	5	0	4.5 ± 1.4	0.41 ± 0.01	0.35 ± 0.01
Corinnidae	*Castianeira cingulata* (Koch)	7	1	1	4.9 ± 2.6	0.17 ± 0.03	0.17 ± 0.02
	Castianeira longipalpa (Hentz)	3	0	0	6.2 ± 0.4	0.39 ± 0.01	0.39 ± 0.02
Dictynidae	*Cicurina brevis* (Emerton)	20	10	36	1.6 ± 0.3	0.19 ± 0.03	0.32 ± 0.02
	Cicurina pallida Keyserling	8	2	17	3.6 ± 0.7	0.26 ± 0.02	0.29 ± 0.04
	Cicurina robusta Simon	2	19	1	4.9 ± 1.6	0.28 ± 0.03	0.25 ± 0.03
	Emblyna sp. 1	1	0	0	0.3	0.11	0.31
	Emblyna sublata (Hentz)	1	1	0	0.4	0.16 ± 0.01	0.35 ± 0.02
	Lathys pallida (Marx)	0	1	0	0.1	0.07	0.3
Gnaphosidae	*Drassyllus fallens* Chamberlin	4	0	1	1.7 ± 0.1	0.15 ± 0.03	0.33 ± 0.09
	Haplodrassus hiemalis (Emereton)	1	0	0	9.9	0.3	0.27
	Herpyllus ecclesiasticus Hentz	1	0	0	4	0.24	0.33
	Zelotes duplex Chamberlain	1	0	0	3.8	0.22	0.32
	Zelotes fratris Chamberlin	0	0	1	5.6	0.33	0.44
	Zelotes hentzi Barrows	1	0	0	4.9	0.31	0.34
Hahniidae	*Antistea brunnea* (Emerton)	0	0	4	0.4	0.09 ± 0.02	0.21 ± 0.03
	Cryphoeca montana Emerton	1	1	467	1.2 ± 0.5	0.15 ± 0.02	0.34 ± 0.05
	Hahnia cinerea Emerton	0	1	0	0.1	0.06	0.23

Hahniidae	*Neoantistea magna* (Keyserling)	255	1033	595	2.1 ± 1	0.17 ± 0.07	0.27 ± 0.05
Linyphiidae	*Agyneta fabra* (Keyserling)	2	0	0	0.2 ± 0.1	0.08 ± 0.03	0.25 ± 0.06
	Agyneta scheffordiana Dupérré & Paquin	8	10	0	0.1	0.06 ± 0.01	0.3
	Agyneta simplex (Emerton)	4	1	0	0.1	0.08	0.34 ± 0.03
	Agyneta sp. 1	2	0	0	0.1	0.07 ± 0.01	0.37 ± 0.05
	Aphileta misera (Pickard-Cambridge)	0	0	9	0.2 ± 0.1	0.07	0.24 ± 0.03
	Bathyphantes brevipes (Emerton)	0	0	1	0.3	0.1	0.23
	Bathyphantes pallidus (Banks)	1	1	111	0.4 ± 0.1	0.09 ± 0.01	0.2
	Bathyphantes weyeri (Emerton)	2	0	0	0.2	0.06	0.13 ± 0.01
	Centromerus persolutus (Pickard-Cambridge)	0	13	47	0.2 ± 0.1	0.1 ± 0.01	0.29 ± 0.03
	Ceraticelus fissiceps (Pickard-Cambridge)	2	0	3	0.1	0.06	0.27
	Ceraticelus laetabilis (Pickard-Cambridge)	2	5	1	0.1 ± 0.1	0.1 ± 0.01	0.43 ± 0.1
	Ceratinella buna Chamberlin	0	3	2	0.1	0.06	0.2
	Cheniseo sphagnicultor Bishop & Crosby	0	0	4	0.1	0.07 ± 0.01	0.3 ± 0.05
	Diplocentria bidentata (Emerton)	0	0	1	0.1	0.06	0.27
	Diplocephalus subrostratus (Pickard-Cambridge)	0	0	18	0.3	0.07	0.17
	Disembolus corneliae (Chamberlin & Ivie)	1	0	0	0	0.06 ± 0.02	0.3 ± 0.11
	Eperigone entomologica (Emerton)	0	0	7	0.1	0.09	0.29 ± 0.04
	Eperigone maculata (Banks)	17	163	268	0.4 ± 0.2	0.13 ± 0.01	0.22 ± 0.01
	Erigone atra Blackwall	3	2	0	0.1	0.05	0.2
	Erigone autumnalis Emerton	0	1	0	0.1	0.07	0.21
	Erigone sp. 1	0	1	0	0.1	0.05	0.23
	Floricomus plumalis (Crosby)	0	4	0	0.1	0.05 ± 0.01	0.3 ± 0.05
	Floricomus praedesignatus (Bishop & Crosby)	0	30	0	0.1	0.05	0.21
	Gnathonaroides pedalis (Emerton)	0	0	1	0.4 ± 0.1	0.12 ± 0.03	0.3 ± 0.08
	Gonatium crassipalpum Bryant	1	2	1	0.1	0.06	0.26 ± 0.02
	Halorates oxypaederotipus (Crosby)	9	70	107	0.1	0.07 ± 0.02	0.25 ± 0.08
	Halorates plumosus (Emerton)	2	0	0	0.9 ± 0.1	0.15 ± 0.02	0.24 ± 0.03
	Helophora insignis (Blackwall)	0	0	4	0.2	0.08	0.21 ± 0.03
	Lepthyphantes alpinus (Emerton)	0	0	31	0.6 ± 0.2	0.12 ± 0.01	0.26 ± 0.05
	Lepthyphantes intricatus (Emerton)	0	7	5	0.4	0.11 ± 0.03	0.26 ± 0.04
	Macrargus multesimus (Pikard-Cambridge)	1	1	0	0.2 ± 0.1	0.06 ± 0.01	0.18 ± 0.02
	Micrargus longitarsus (Emerton)	0	0	6	0.3 ± 0.1	0.09 ± 0.01	0.25 ± 0.04
	Microneta viaria (Blackwall)	11	0	4	1.2	0.13 ± 0.01	0.19 ± 0.01
Linyphiidae	*Oreonetides vaginatus* (Thorell)	0	0	3	0.7 ± 0.1	0.12	0.2 ± 0.04
	Oreophantes recurvatus (Emerton)	0	0	2	2.2 ± 0.7	0.24 ± 0.02	0.26 ± 0.02
	Pityophantes subarcticus Chamberlin & Ivie	0	1	4	0.2	0.07 ± 0.01	0.26 ± 0.04

	Pocadicnemis americana Millidge	0	26	72	0.1	0.08 ± 0.03	0.27 ± 0.05
	Porrhomma terrestre (Emerton)	0	1	1	0.1	0.06	0.25
	Sciastes truncatus (Emerton)	0	0	1	0.2	0.1	0.24
	Scironis tarsalis (Emerton)	0	0	28	0.2	0.05	0.21 ± 0.04
	Sisicottus montanus (Emerton)	0	0	7	0	0.06 ± 0.01	0.3 ± 0.04
	Sisicus penifusifer Bishop & Crosby	0	3	2	0.1	0.08 ± 0.01	0.34 ± 0.04
	Soulgas corticarius (Emerton)	0	0	4	0	0.04 ± 0.01	0.28 ± 0.08
	Tapinocyba minuta (Emerton)	0	0	5	0.1	0.05	0.25
	Tapinocyba simplex (Emerton)	1	0	11	0.3	0.08	0.2 ± 0.03
	Tenuiphantes zebra (Emerton)	2	44	41	0.2	0.06	0.21
	Tunagyna debilis (Banks)	0	0	114	0.3 ± 0.1	0.07 ± 0.02	0.18 ± 0.04
	Walckenaeria atrotibialis (Pickard-Cambridge)	26	102	37	0.4 ± 0.1	0.07 ± 0.03	0.16 ± 0.06
	Walckenaeria castanea (Emerton)	0	0	24	0.5 ± 0.1	0.09 ± 0.01	0.22 ± 0.02
	Walckenaeria communis Emerton	0	0	11	0.2 ± 0.1	0.08 ± 0.02	0.21 ± 0.02
	Walckenaeria digitata (Emerton)	7	0	1	0.3	0.08 ± 0.01	0.26 ± 0.01
	Walckenaeria directa (Pickard-Cambridge)	1	18	54	0.1	0.07 ± 0.02	0.31 ± 0.03
	Walckenaeria exigua Millidge	4	0	4	0.3	0.1	0.31
	Walckenaeria spiralis (Emerton)	0	1	0	0.4	0.09	0.26 ± 0.01
	Wubana pacifica (Banks)	0	0	3	0.1 ± 0.1	0.07	0.32 ± 0.02
	Unknown sp. 1	0	1	0	0.1	0.05 ± 0.01	0.23
	Unknown sp. 2	0	0	1	0.2	0.08	0.27
	Unknown sp. 3	0	1	0	0.1	0.07	0.3
	Unknown sp. 4	0	0	1	3.7 ± 0.8	0.22 ± 0.02	0.28 ± 0.02
Liocranidae	*Agroeca ornata* Banks	14	16	0	9	0.39	0.35
Lycosidae	*Geolycosa domifex* (Hancock)	0	0	1	45.4	0.77	0.42
	Gladicosa gulosa (Walckenaer)	1	0	0	6.6	0.35	0.33
	Pardosa labradorensis (Thorell)	0	0	1	20	0.7	0.47
	Pardosa mackenziana (Keyserling)	0	0	15	6.3 ± 2	0.34 ± 0.06	0.34 ± 0.04
	Pirata montanus Emerton	168	153	0	3.2 ± 0.6	0.22 ± 0.06	0.3 ± 0.06
	Pirata piraticus (Clerck)	1	0	0	3.3	0.16	0.24
	Piratula insularis (Emerton)	0	0	1	2.2	0.16	0.24
Lycosidae	*Trochosa ruricola* (DeGeer)	1	0	0	46.6	0.99	0.44
	Trochosa terricola Thorell	2	0	0	23.8 ± 4.5	0.67 ± 0.08	0.37 ± 0.06
Philodromidae	*Philodromus exilis* Banks	1	1	1	1.5 ± 0.4	0.22 ± 0.03	0.45 ± 0.01
	Philodromus praelustris Keyserling	0	1	0	5.7	0.45	0.51
Phrurolithidae	*Phrurotimpus alarius* (Hentz)	199	138	0	0.4 ± 0.1	0.11 ± 0.01	0.37 ± 0.05
	Phrurotimpus borealis (Emerton)	47	0	0	0.5 ± 0.2	0.19	0.63

Salticidae	*Maevia inclemens* (Walckenaer)	1	0	0	3.2	0.32	0.56
	Neon nellii Peckham & Peckham	3	4	5	0.6 ± 0.2	0.13 ± 0.01	0.54 ± 0.18
	Pelegrina proterva (Walckenaer)	0	1	0	1	0.15	0.29
Tetragnathidae	*Meta ovalis* (Gertsch)	0	1	0	21.4	0.43	0.32
Theridiidae	*Canalidion montanum* (Emerton)	0	0	1	0.4	0.12	0.36
	Robertus fuscus (Emerton)	0	0	1	0.5	0.34	0.33
	Robertus pumilus (Emerton)	1	0	0	0.2	0.1 ± 0.01	0.28 ± 0.01
	Robertus riparius (Keyserling)	0	5	182	1.1 ± 0.2	0.18 ± 0.04	0.3 ± 0.05
	Rugathodes sexpunctatus (Emerton)	0	1	11	0.2 ± 0.1	0.1 ± 0.01	0.36 ± 0.07
	Steatoda borealis (Hentz)	0	0	1	3.4 ± 1	0.33 ± 0.07	0.5 ± 0.07
	Theridion montanum Emerton	0	0	1	0.3	0.14	0.5
Thomisidae	*Ozyptila distans* Dondale & Redner	4	1	0	2.4 ± 0.7	0.23	0.45
	Xysticus elegans Keyserling	16	19	0	19.5 ± 9.2	0.56 ± 0.02	0.56 ± 0.02
**Opiliones**							
Caddidae	*Caddo agilis* Banks	1	0	0	0.8	0.16	0.33
Ceratolasmatidae	*Crosbycus dasycnemus* (Crosby)	0	1	0	0.1	0.04	0.25
Phalangiidae	*Mitopus morio* (Faricius)	0	0	1	3.7	0.24	0.24
	Unknown sp. 1	0	0	18	5.4 ± 3.7	0.24 ± 0.05	0.29 ± 0.02
	Odiellus pictus (Wood)	17	57	202	5.4 ± 3.1	0.26 ± 0.06	0.31 ± 0.04
	Platybunus ? sp.	6	0	0	0.1	0.09 ± 0.02	0.33 ± 0.03
	Rilaena triangularis (Herbst)	5	3	0	8.3 ± 5.2	0.24 ± 0.05	0.24 ± 0.06
Sabaconidae	*Sabacon cavicolens* (Packard)	0	0	1	0.7	0.14	0.33
Sclerosomatidae	*Hadrobunus maculosus* (Wood)	70	23	34	3.9	0.21	0.29
	Leiobunum aldrichi (Weed)	24	20	0	5.7 ± 1.5	0.19 ± 0.02	0.26 ± 0.03
	Leiobunum bracchiolum McGhee	478	88	0	4.5 ± 2.8	0.21 ± 0.02	0.32 ± 0.03
	Leiobunum calcar (Wood)	56	86	28	14 ± 2.7	0.3 ± 0.05	0.28 ± 0.04
	Leiobunum elegans(?) Weed	0	0	1	8.2	0.25	0.27
	Leiobunum flavum Davis	4	8	10	20.1 ± 6.7	0.31 ± 0.02	0.3 ± 0.02
Sclerosomatidae	*Leiobunum politum* Weed	25	8	0	2.1 ± 0.3	0.18 ± 0.05	0.3 ± 0.06
	Leiobunum ventricosum (Wood)	0	17	22	12.2 ± 2.1	0.28 ± 0.04	0.28 ± 0.04
Trogulidae	*Trogulus tricarinatus* (Linné)	0	14	0	3.3 ± 0.6	0.09 ± 0.01	0.19 ± 0.02

Table S4 Abundance and range of functional trait values (± standard error) of Diplopoda and Isopoda caught in pitfall traps in three forests of southern Québec, Canada. Details on trait measurements are provided in Appendix S1. SB = Mont-St-Bruno, SH = Mont-St-Hilaire, MÉ = Mont-Écho.

		Abundance			Functional traits value					
**Family**	**Species**	**SB**	**SH**	**MÉ**	**Body volume (mm^3)**	**Biting force index**	**Molar plate force index**	**Mandibles gape (mm)**	**Cuticular toughness ($g\ mm^{-2}$)**	**Body width (mm)**
**Diplopoda**										
Blaniulidae	*Blaniulus guttulatus* (Fabricius)	6	0	0	2.9 ± 0.8	0.3 ± 0.01	0.23 ± 0.04	0.12 ± 0.03	2	0.55 ± 0.05
	Nopoiulus kochii (Gervais)	17	0	0	2.2 ± 0.7	0.38 ± 0.04	0.24 ± 0.02	0.12 ± 0.02	2	0.55 ± 0.04
Caseyidae	*Underwoodia iuloides* (Harger)	139	0	0	29.7 ± 7.9	0.78 ± 0.07	0.68 ± 0.04	0.26 ± 0.03	2	1.47 ± 0.2
Cleidogonidae	*Cleidogona* sp.	2	1	5	3.1 ± 2.7	0.46 ± 0.11	0.38 ± 0.06	0.12 ± 0.03	0	0.64 ± 0.19
Julidae	*Cylindroiulus caeruleocinctus* (Wood)	572	0	0	72.3 ± 39.3	1.26 ± 0.22	0.46 ± 0.04	0.42 ± 0.06	18 ± 8	1.98 ± 0.33
	Cylindroiulus latestriatus (Curtis)	111	0	0	8.3 ± 4.3	0.62 ± 0.04	0.33 ± 0.04	0.24 ± 0.03	2 ± 1	0.92 ± 0.18
	Ophyiulus pilosus (Newport)	332	2233	0	26.9 ± 14.1	0.82 ± 0.13	0.38 ± 0.05	0.29 ± 0.03	8 ± 5	1.3 ± 0.24
Okeanobatidae	*Okeanobates americanus* Enghoff	1	0	0	2.5	0.24	0.23	0.23	4	0.6
Parajulidae	*Oriulus venustus* (Wood)	32	1	0	135.6 ± 40.4	1.11 ± 0.06	0.66 ± 0.03	0.38 ± 0.05	9 ± 6	2.19 ± 0.25
	Uroblaniulus canadensis (Newport)	256	139	606	91.4 ± 14.6	0.97 ± 0.08	0.69 ± 0.03	0.34 ± 0.04	13 ± 9	1.88 ± 0.18
Polydesmidae	*Polydesmus inconstans* Latzel	1059	11	0	12.5 ± 2.4	0.55 ± 0.02	0.63 ± 0.03	0.24 ± 0.02	2 ± 1	1.37 ± 0.17
	Pseudopolydesmus canadensis Newport	0	0	3	148.8 ± 8.5	1.27	1.15	0.38	14	3.35 ± 0.07
	Pseudopolydesmus serratus (Say)	129	985	178	143.1 ± 20	1.27 ± 0.1	1.12 ± 0.08	0.48 ± 0.04	9 ± 10	3.42 ± 0.31
Spirobolidae	*Narceus americanus* (de Beauvois)	354	23	0	2111.8 ± 460	3.46 ± 0.26	1.18 ± 0.09	1.38 ± 0.12	185 ± 53	5.65 ± 0.42
Trichopetalidae	*Trichopetalum lunatum* Harger	9	9	31	1.7 ± 0.2	0.32 ± 0.06	0.31 ± 0.02	0.14 ± 0.02	0	0.57 ± 0.03
	Unknown sp. 1	15	7	1	1.4	0.42	0.28	0.09	0	0.55
**Isopoda**										
Oniscidae	*Oniscus asellus* Linné	59	140	0	55.9 ± 35.1	1.44 ± 0.33	0.57 ± 0.21	0.52 ± 0.07	17 ± 5	5.9 ± 0.98
Trachelipodidae	*Trachelipus rathkei* (Brandt)	1366	0	0	40.4 ± 9.5	1.46 ± 0.2	0.45 ± 0.06	0.49 ± 0.08	13 ± 2	5.18 ± 0.45
Trichoniscidae	*Hyloniscus riparius* (Koch)	182	4	0	1.6 ± 0.7	0.42 ± 0.02	0.2 ± 0.07	0.23 ± 0.06	0	1.75 ± 0.32
	Trichoniscus pusillus Brandt	309	61	0	0.6 ± 0.1	0.34 ± 0.04	0.11 ± 0.03	0.21 ± 0.05	0	1.26 ± 0.12

Table S5 Abundance and range of functional trait values (± standard error) of larval insects (representing potential prey) caught in pitfall traps in three forests of southern Québec, Canada. Details on trait measurements are provided in Appendix S1. SB = Mont-St-Bruno, SH = Mont-St-Hilaire, MÉ = Mont-Écho. ~ = near (meaning the identification is unsure, but is a close relative of this genus).

		Abundance			Functional trait value		
**Famille**	**Species**	**SB**	**SH**	**MÉ**	**Body volume (mm^3)**	**Cuticular toughness ($g\ mm^{-2}$)**	**Body width (mm)**
**Coleoptera**							
Cantharidae	Cantharidae sp. 1	2	55	114	6.8 ± 6.9	4 ± 3	1.08 ± 0.42
	Cantharidae sp. 2	0	1	1	0.2 ± 0.1	0	0.3 ± 0.05
	Cantharidae sp. 3	0	8	14	1.9 ± 0.6	2	0.78 ± 0.03
	Cantharidae sp. 4	0	5	23	21.6 ± 25.3	10	1.68 ± 0.56
Chrysomelidae	Chrysomelidae sp. 1	1	2	0	8.9 ± 6.3	23 ± 6	1.68 ± 0.41
	Chrysomelidae sp. 2	6	0	0	5.9 ± 3.5	0	1.16 ± 0.34
Curculionidae	Curculionidae sp. 1	1	4	0	2 ± 0.2	0	0.9 ± 0.07
Elateridae	*Limonius* sp.	0	2	1	40.2 ± 45	14 ± 13	1.87 ± 0.93
	Elateridae sp. 1	0	2	4	3.2 ± 3.6	8	0.61 ± 0.23
Endomychidae	Endomychidae sp.	2	0	0	10.8 ± 9	17 ± 1	1.15 ± 0.35
Lampyridae	*Lucidota atra* (Olivier)	54	85	47	19.7 ± 12	2 ± 2	1.54 ± 0.54
	Pyractomena sp.	0	21	1	32.2 ± 32.5	2 ± 2	2.33 ± 1.43
Latridiidae	Latridiidae sp.	1	0	0	0.1 ± 0.1	0	0.31 ± 0.01
Leiodidae	Cholevinae sp.	1	2	2	0.1	0	0.3 ± 0.02
Lycidae	Lycidae sp.	4	8	17	6.4 ± 7.3	2 ± 1	1.11 ± 0.32
Melandryidae	Melandryidae sp.	0	2	0	2.6 ± 3.4	0	0.64 ± 0.51
Meloidae	*Meloe* sp.	4	2	0	0.1	1	0.28 ± 0.02
Ptiliidae	Ptiliidae sp.	1	0	9	0.1	0	0.19 ± 0.04
Staphylinidae	*Gabrius* sp.	0	0	3	0.8 ± 0.5	1	0.52 ± 0.12
	Philonthus sp.	5	0	3	3.5 ± 4.5	1	0.81 ± 0.43
	Tachinus sp.	1	3	59	4 ± 5.3	1 ± 1	0.87 ± 0.25
	~*Tasgius* sp.	17	1	0	53.4 ± 5.5	14	2.18 ± 0.11
	Aleocharinae sp.	0	0	1	0.1 ± 0.1	0	0.28 ± 0.08
	Oxytelinae sp.	9	2	13	1 ± 0.8	0	0.61 ± 0.22
	Tachininae sp.	1	7	22	3.6 ± 1.6	1	0.9 ± 0.16
	Staphylinidae sp. 1	0	0	2	0.2 ± 0.1	1 ± 1	0.35 ± 0.04
	Staphylinidae sp. 2	1	0	1	0.3 ± 0.2	1	0.38 ± 0.05
	Staphylinidae sp. 3	2	4	6	0.1	1	0.32 ± 0.04
	Staphylinidae sp. 4	0	0	11	3.3 ± 0.8	1	0.79 ± 0.08
Tenebrionidae	Tenebrionidae sp.	7	0	0	16.7 ± 28.7	7 ± 7	1.18 ± 0.7
Unknown	Coleoptera sp. 1	0	2	5	0.4 ± 0.4	0 ± 1	0.39 ± 0.16
	Coleoptera sp. 2	1	0	0	0.2 ± 0.1	0	0.48 ± 0.02
	Coleoptera sp. 3	3	0	0	0.3	0	0.36 ± 0.01
**Diptera**							
Bibionidae	*Bibio* sp. 1	0	2	9	13 ± 3.6	3 ± 3	1.3 ± 0.15
	Bibio sp. 2	1	2	2	12.2 ± 10.1	7 ± 2	1.35 ± 0.37
	Plecia sp.	0	0	4	32.9 ± 25	7 ± 1	1.75 ± 0.47
Calliphoridae	Calliphoridae sp.	0	0	4	1.8 ± 0.1	0	0.73 ± 0.04
Cecidomyiidae	~*Lestodiplosis* sp.	1	3	3	0.2 ± 0.2	0	0.3 ± 0.12
	Cecidomyiidae sp. 1	1	0	1	0.5 ± 0.5	0	0.45 ± 0.17
	Cecidomyiidae sp. 2	0	1	7	0.1	0	0.22 ± 0.03
	Cecidomyiidae sp. 3	0	0	5	0.5 ± 0.4	0	0.5 ± 0.14
	Cecidomyiidae sp. 4	1	0	4	0.6 ± 0.2	0	0.54 ± 0.09
	Cecidomyiidae sp. 5	0	2	1	0.1 ± 0.1	0	0.26 ± 0.12

Cecidomyiidae	Cecidomyiidae sp. 6	0	1	5	0.2 ± 0.1	0	0.33 ± 0.09
	Cecidomyiidae sp. 7	2	2	0	1.6 ± 2	0	0.65 ± 0.32
	Cecidomyiidae sp. 8	0	2	7	0	0	0.18 ± 0.03
	Cecidomyiidae sp. 9	0	11	1	0.1 ± 0.1	0	0.3 ± 0.08
	Cecidomyiidae sp. 10	3	4	3	0.5 ± 0.2	0	0.5 ± 0.08
	Cecidomyiidae sp. 11	0	3	0	0.1	0	0.31 ± 0.02
	Cecidomyiidae sp. 12	0	1	3	0.2 ± 0.1	0	0.38 ± 0.06
Chironomidae	Chironomidae sp. 1	0	2	2	0.2 ± 0.1	0	0.28 ± 0.09
	Chironomidae sp. 2	0	0	3	1.3 ± 0.8	0	0.49 ± 0.13
	Chironomidae sp. 3	0	1	2	0.4 ± 0.3	0	0.33 ± 0.11
Drosophilidae	Drosophilidae sp. 1	1	21	28	1.2 ± 0.6	0	0.63 ± 0.11
	Drosophilidae sp. 2	0	0	5	0.7 ± 0.6	0	0.53 ± 0.18
Empididae	Empididae sp.	0	0	2	1.4 ± 1.8	0	0.55 ± 0.33
Fanniidae	Fanniidae sp.	0	0	6	5.4 ± 4.8	1 ± 1	1.03 ± 0.6
Limoniidae	*Hexatoma* sp.	0	0	12	6.9 ± 4.1	3 ± 2	0.92 ± 0.25
	Ormosia sp.	0	4	15	2.1 ± 2.2	5 ± 1	0.65 ± 0.22
	Limoniidae sp. 1	1	0	0	13 ± 13.8	4 ± 4	1.13 ± 0.53
	Limoniidae sp. 2	0	0	1	2.5 ± 2.3	1 ± 2	0.71 ± 0.25
	Limoniidae sp. 3	1	0	3	5 ± 0.9	3 ± 1	0.95 ± 0.08
	Limoniidae sp. 4	0	0	1	1.1 ± 1	0	0.51 ± 0.19
	Limoniidae sp. 5	2	3	0	19.8 ± 14.2	1 ± 2	1.5 ± 0.34
	Limoniidae sp. 6	0	0	2	4.8 ± 3.8	1 ± 1	0.83 ± 0.39
Muscidae	Muscidae sp.	0	0	13	0.3 ± 0.1	0	0.39 ± 0.05
Mycetophilidae	Mycetophilidae sp.	0	4	3	9.4 ± 3.4	1 ± 1	1.24 ± 0.14
Pediciidae	*Pedicia* sp.	4	7	8	3.4 ± 0.5	1 ± 1	0.84 ± 0.05
Phoridae	~*Megaselia* sp.	5	5	5	1.2 ± 0.5	0	0.67 ± 0.09
	Phoridae sp. 1	0	1	4	0.3 ± 0.2	0	0.44 ± 0.07
	Phoridae sp. 2	0	0	1	5 ± 3.4	0	1.08 ± 0.29
	Phoridae sp. 3	0	1	0	2.4 ± 1.9	0	0.8 ± 0.26
Psychodidae	Psychodidae sp. 1	0	0	3	0.2 ± 0.1	0	0.33 ± 0.07
	Psychodidae sp. 2	0	0	6	0.4 ± 0.1	0	0.41 ± 0.07
Sarcophagidae	Sarcophagidae sp.	6	10	0	0.7 ± 0.4	0	0.57 ± 0.14
Sciaridae	Sciaridae sp. 1	1	1	17	1.3 ± 0.8	1 ± 1	0.52 ± 0.17
	Sciaridae sp. 2	0	0	1	0.1	0	0.18 ± 0.02
Therevidae	Therevidae sp.	1	0	0	11.4 ± 11.1	5 ± 1	0.85 ± 0.29
Tipulidae	*Tipula* sp. 1	0	3	1	32.8 ± 16.8	1 ± 1	1.86 ± 0.39
	Tipula sp. 2	0	0	1	37.4 ± 12.8	5 ± 4	2 ± 0.14
	Tipula sp. 3	0	0	3	55.4 ± 52.9	2	2.11 ± 0.93
	Tipula sp. 4	1	8	0	169.9 ±136.5	9 ± 5	2.93 ± 1.05
	Tipula sp. 5	0	0	4	58.6 ± 44.5	5 ± 1	2.14 ± 0.77
Unknown	Diptera sp. 1	2	1	1	7.1 ± 3.6	2 ± 3	1.13 ± 0.16
	Diptera sp. 2	0	4	0	3.9 ± 0.5	0	1.16 ± 0.12
	Diptera sp. 3	0	1	1	3.2	0	1
	Diptera sp. 4	2	1	2	0.2 ± 0.1	0	0.33 ± 0.05
	Diptera sp. 5	0	0	1	5 ± 8.3	0	0.69 ± 0.62
	Diptera sp. 6	0	0	14	4.8 ± 1.9	0	1.15 ± 0.19
**Lepidoptera**							
Adelidae	Adelidae sp.	1	1	3	41.4 ± 22.5	10 ± 5	3.6 ± 0.76
Erebidae	Erebidae sp.	3	3	0	314.7 ±142.7	3 ± 1	3.75 ± 0.64
Gelechiidae	Gelechiidae sp. 1	1	4	0	10.1 ± 4.3	2 ± 1	1.2 ± 0.17
	Gelechiidae sp. 2	1	3	0	35.9 ± 8	6	1.85 ± 0.17
Noctuidae	Noctuidae sp. 1	14	4	6	41.6 ± 107.5	2 ± 2	1.03 ± 1.14
	Noctuidae sp. 2	3	0	0	15.2 ± 15.2	4 ± 4	1.31 ± 0.44
	Noctuidae sp. 3	15	67	14	264.8 ±238.2	7 ± 5	2.95 ± 1.64
	Noctuidae sp. 4	0	0	4	103.7 ±104.4	2 ± 3	2.28 ± 0.88
	Noctuidae sp. 5	1	0	3	28 ± 15.3	2 ± 3	1.63 ± 0.32

	Noctuidae sp. 6	2	0	7	52.2 ± 94.9	1 ± 3	1.66 ± 1.14
	Noctuidae sp. 7	0	0	5	303.1 ±164.5	4 ± 3	3.31 ± 1.08
	Noctuidae sp. 8	0	1	0	13.4 ± 8	4	1.3 ± 0.36
	Noctuidae sp. 9	19	5	2	126.7 ±117.2	1 ± 1	2.79 ± 0.75
Psychidae	*Dahlica* sp.	68	41	1	0.5 ± 0.3	10	0.41 ± 0.09
	Psyche sp.	141	51	7	3.9 ± 9.1	20 ± 6	0.57 ± 0.75
Pyralidae	Pyralidae sp.	2	0	1	0.1 ± 0	0	0.2 ± 0.04
Tortricidae	Tortricidae sp. 1	10	10	7	7.6 ± 2.8	0	1.03 ± 0.13
	Tortricidae sp. 2	0	6	0	7.9 ± 3.7	1 ± 1	1.05 ± 0.19
	Tortricidae sp. 3	6	0	0	114.5 ±139.1	1 ± 1	2.06 ± 1.17
Unknown	Lepidoptera sp. 1	19	3	2	0.4 ± 0.4	0	0.35 ± 0.18
	Lepidoptera sp. 2	0	2	0	4	0	0.85
	Lepidoptera sp. 3	0	1	0	2.2 ± 3	2 ± 3	0.59 ± 0.28
	Lepidoptera sp. 4	0	0	18	33.4 ± 26	3 ± 1	1.41 ± 0.44
**Mecoptera**							
Panorpidae	*Panorpa* sp. 1	5	2	86	6.8 ± 7.6	7 ± 1	0.92 ± 0.38
	Panorpa sp. 2	1	4	0	22.8 ± 19.2	8	1.41 ± 0.68
